# CORE-HPE Reporting Guidelines: Standardizing Reporting for Competency-Based Health Professions Education Outcomes Research

**DOI:** 10.5334/pme.2554

**Published:** 2026-09-29

**Authors:** Aleda M. H. Chen, Denise H. Rhoney, Susan Humphrey-Murto, Mary Ellen J. Goldhamer, Linda Snell, Holly Caretta-Weyer, Daniel J. Schumacher, Soeren Huwendiek, Arvin Damodaran, Andrew K. Hall, Robert Englander, Laura Molgaard, Anna Oswald, Pedro Tanaka, Fremen Chihchen Chou, Jason R. Frank, as members of the International Competency-based Education in the Health Professions Collaborators

**Affiliations:** 1 University of Arkansas for Medical Sciences, Little Rock, Arkansas, USA; 2 UNC Eshelman School of Pharmacy, Chapel Hill, NC, USA; 3 University of Ottawa, Ottawa, Canada; 4 University of Ottawa, The Ottawa Hospital, Ottawa, Canada; 5 Division of General Internal Medicine, Massachusetts General Hospital, Boston, Massachusetts, USA; 6 Harvard Kidney Urology Hematology Training Institute, Boston, Massachusetts, USA; 7 McGill University, Montreal, Canada; 8 Royal College of Physicians and Surgeons of Canada, Ottawa, Canada; 9 Stanford University School of Medicine, Palo Alto, California, USA; 10 Cincinnati Children’s Hospital Medical Center/University of Cincinnati College of Medicine, Cincinnati, Ohio, USA; 11 Department for Assessment and Evaluation, Institute for Medical Education, University of Bern, Bern, Switzerland; 12 School of Clinical Medicine, Faculty of Medicine and Health, UNSW, Sydney, Australia; 13 Department of Emergency Medicine, University of Ottawa, Ottawa, Canada; 14 Department of Medical Education, University of Illinois College of Medicine, Chicago, IL, USA; 15 University of Minnesota College of Veterinary Medicine, St. Paul, Minnesota, USA; 16 Department of Medicine, University of Alberta, Edmonton, Canada; 17 Stanford University School of Medicine, Stanford, California, USA; 18 China Medical University Hospital, Taiwan; 19 School of Medicine, China Medical University, Taichung, Taiwan; 20 The Ottawa Hospital, Ottawa, Canada

## Abstract

**Background:**

Competency-Based Health Professions Education (CBHPE) is increasingly implemented; however, CBHPE outcomes research remains difficult to interpret and synthesize when studies incompletely report the CBHPE model and other elements.

**Objective:**

To develop structured reporting guidelines for CBHPE outcomes studies to improve transparency, interpretability, and transferability across educational contexts and professions.

**Methods:**

A multi-phase, iterative consensus-development process was used to create a CBHPE outcomes reporting checklist aligned with a CBHPE Outcomes Theory of Change. Draft reporting elements were generated and refined through structured writing-group deliberations, broad stakeholder feedback gathered through interactive town hall sessions within the ICBHPE Collaborative, and iterative revision cycles. The checklist was then pilot-tested with non-expert users to assess clarity, feasibility, and usability, and revisions were made to reduce redundancy, harmonize terminology, and strengthen guidance for high-impact reporting domains.

**Results:**

The final product is a structured reporting checklist, organized by manuscript section, accompanied by brief item descriptions and rationales, and registered with EQUATOR. The guidelines prioritize reporting elements that support causal interpretability in CBHPE outcomes research, including specification of the CBHPE intervention architecture and core components, program theory and intended mechanisms, implementation fidelity and adaptations, assessment system design and governance for progression decisions, multi-level outcomes and indicators across developmental timeframes, contextual enablers and constraints, and equity considerations.

**Conclusions:**

These CBHPE outcomes reporting recommendations provide a practical tool and guideline for authors, reviewers, and editors to strengthen the completeness and consistency of CBHPE outcomes reporting and should be considered before beginning a study. Adoption of this checklist may improve interpretability and evidence synthesis across programs and accelerate actionable learning about what works in CBHPE, for whom, in what contexts, and why.

## Introduction

Competency-Based Medical Education (CBME) was introduced in medical education after the World Health Organization called for it in 1978 [1]. More recently, this has expanded to include other health professions. Competency-based health professions education (CBHPE) is an outcomes-oriented approach to training that ensures learners progressively develop and demonstrate competence in authentic clinical and professional contexts [2]. Rather than relying primarily on time-based advancement, CBHPE emphasizes achievement of transparent outcome competencies, sequenced developmental expectations, meaningful workplace learning through competency-focused instruction, individualized learning, and assessment programs intended to support longitudinal progression decisions [2]. These five CBHPE core components are an essential foundation for the reporting guidelines detailed in this paper. A central premise of CBHPE is that learner development can be strengthened when training experiences, coaching, assessment, and promotion decisions are aligned around competence that is visible, measurable, and relevant to patient, community, and learner needs [2].

Over the past decade, CBHPE implementation has expanded rapidly across health professions and the educational continuum, reflecting increasing expectations for educational accountability, practice readiness, and alignment with societal needs [3,4,5,6,7,8,9,10,11,12,13,14,15]. The authors refer readers to two additional papers in this PME supplement, one that focuses on the evaluation of CBHPE programs around the globe [16] and another that describes how CBHPE programs have evolved and adapted over time [17]. Examples include CBHPE in single programs [18,19,20], single specialty [21,22,23,24], single institution [25], national implementation in Canada and Taiwan [26,27,28,29], and multi-country efforts (Switzerland, the Netherlands, Finland) [30]. These papers highlight numerous studies detailing the depth and breadth of CBHPE program development, implementation, evolution, adaptation, and evaluation, providing a roadmap for reporting CBHPE innovations. However, CBHPE is not a single intervention; it is a complex, system-level educational transformation composed of multiple interacting components, stakeholder behaviors, and infrastructure requirements [31]. This complexity creates persistent challenges for outcomes research and evaluation because improvements in learner performance, clinical care, or system outcomes may be shaped as much by implementation context and fidelity as by the educational model itself. In their critical narrative review, Brydges and colleagues highlighted that the evidence base underpinning competency-based approaches is substantial but mixed, and they emphasized the need for the field to make assumptions explicit and generate stronger evidence about how and why these models work (or do not) across contexts [32].

In parallel, the CBHPE literature reflects considerable heterogeneity in how CBHPE is conceptualized, justified, and operationalized. Hamza and colleagues examined how competency-based approaches are discussed across the health professions education literature. They identified recurring themes related to perceived benefits and drawbacks, skepticism and uncertainty, implementation complexity, and proposed pathways for advancing the field [33]. Their work reinforces that incomplete reporting, such as variation in design and implementation and inconsistency in how interventions and outcomes are reported, continues to limit interpretability and slow the accumulation of actionable evidence [16,33]. Incomplete reporting in CBHPE scholarship limits interpretation, reproducibility, and evidence synthesis, and this contributes to conflicting conclusions about effectiveness - concerns that have been documented across health professions education research and systematic reviews [16,31,34,35]. For example, studies may report isolated elements or core components of CBHPE implementation, such as a competency framework or new assessment methods, without sufficiently describing the broader CBHPE architecture, including how competencies are sequenced, how workplace learning experiences are structured, how coaching is operationalized, and how assessment data are synthesized into progression decisions. Without system-level context, outcomes cannot be meaningfully attributed to CBHPE, nor can differences between programs be interpreted as true effects versus differences in design, implementation fidelity, or context.

The problem of incomplete reporting is especially consequential in CBHPE because outcomes are expected to unfold across learning levels and training continuums. Hall and colleagues’ CBHPE outcomes taxonomy highlights that CBHPE outcomes are inherently multi-level and temporally distributed, including by focus (educational versus clinical), level (micro/meso/macro), and timeline (training, transition to practice, practice) [36]. Fidelity is particularly important in complex educational innovations because outcomes may be influenced by the degree to which core elements are present, the quality of enactment, participant engagement, and differentiation from prior practices. When fidelity is not described, it becomes difficult to determine whether null findings reflect an ineffective educational theory (the model does not work) or an implementation gap (the model was not implemented in a way that could plausibly produce desired change or was not implemented as intended), or some combination of the two. CBHPE implementation also may vary systematically based on local capacity, faculty development infrastructure, workplace-based assessment system validity and bias [37], assessment culture, and organizational readiness, potentially confounding observed outcomes. This further underscores the need for clearer, more standardized reporting to support meaningful evaluation and continuous improvement.

Several methodological and reporting frameworks provide useful precedent for strengthening transparency in educational research. For example, the Template for Intervention Description and Replication (TIDieR) provides structured expectations for reporting interventions in sufficient detail to support replication [38]. In the competency-based domain, the Competency Framework for Development - Health Professions (CONFERD-HP) offers reporting recommendations for developing competency frameworks [39]. However, these frameworks do not fully address all five core components [2]. It is important to close these gaps in the reporting of the implementation of the core components of CBHPE [40]. Specifically, the interpretation of outcomes depends on clear documentation of the CBHPE system architecture (e.g., coaching models, workplace-based assessment structures, governance, and progression decision processes), implementation supports and adaptations, contextual enablers and constraints, and the indicators and metrics used to monitor both proximal outputs and longer-term outcomes. Program theory suggests that it is important to elucidate how what is implemented (theory of action) relates to how outcomes are expected to occur (theory of change) [41]. Thus, CBHPE outcomes research would benefit from purpose-built reporting recommendations that are aligned with program theory. Clear reporting expectations can strengthen interpretability, reduce misattribution of outcomes, facilitate cross-study comparison, and support the field’s ability to accumulate actionable evidence about what works, for whom, in what contexts, and why. Thus, the purpose of this article is to develop structured reporting guidelines for CBHPE outcomes studies to improve transparency, reproducibility, and evidence synthesis in this rapidly evolving domain.

## Methods

### Design and Development Process

A multi-phase, iterative consensus-development process was used to generate reporting recommendations for CBHPE outcomes studies. The reporting guideline process was submitted to EQUATOR (Enhancing the QUAlity and Transparency Of health Research) [42] before consensus and post-completion to support visibility, dissemination, and standardized use across journals and professional contexts. See Figure 1 for an overview of the process of development.

**Figure 1:**
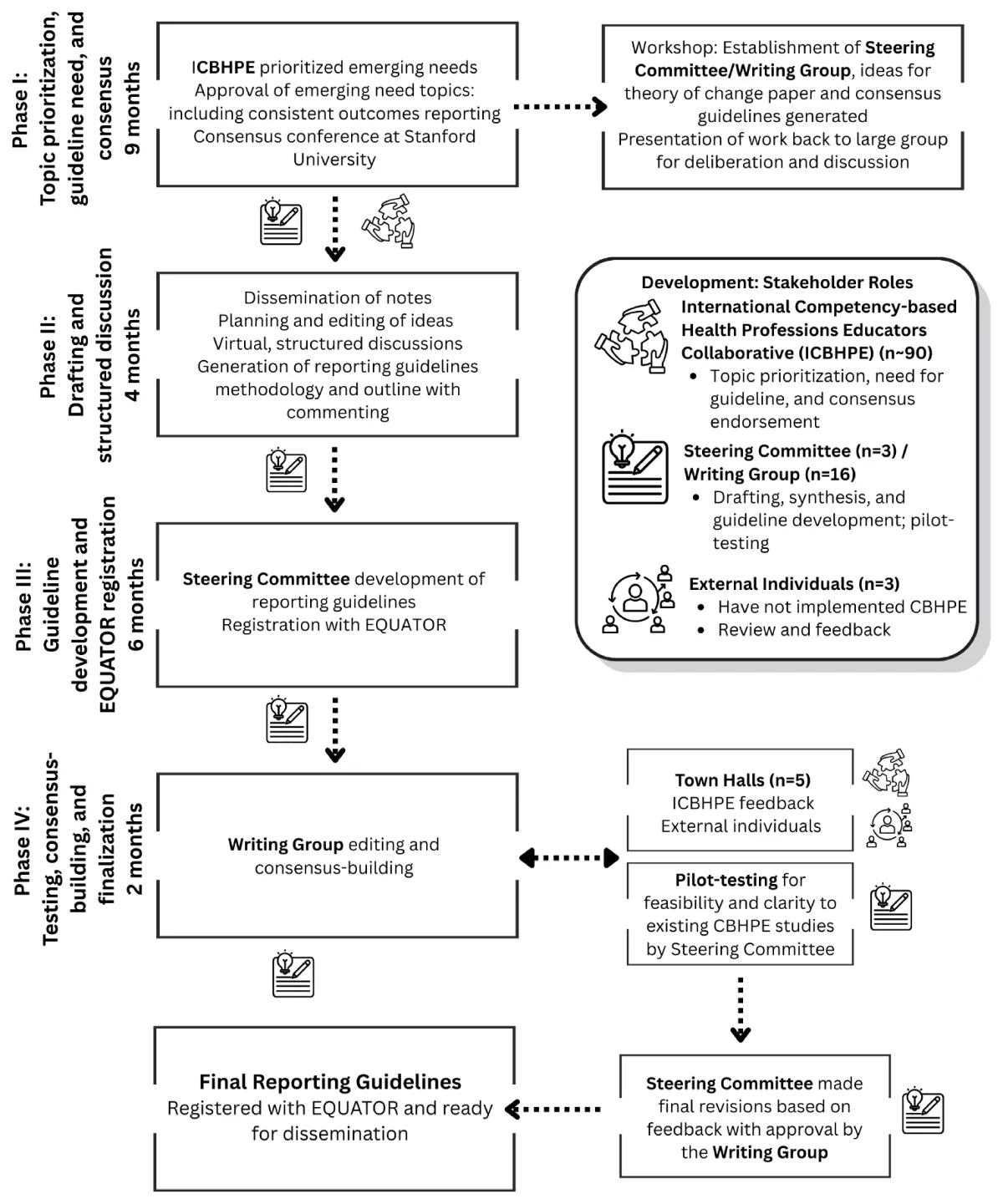
Overview Of Reporting Guidelines Development And Consensus Process.

The consensus-development process for this reporting guideline was structured to emphasize transparency and reproducibility [43]. The development team used EQUATOR to support systematic processes for stakeholder engagement, iterative refinement, and usability testing [44]. In addition, checklist development was informed by published recommendations for Delphi reporting quality (CREDES) and broader consensus reporting guidance (ACCORD) to ensure that consensus processes were described in a complete and interpretable manner [45,46]. The consensus process occurred in two primary stages: (1) development and refinement of draft reporting elements through structured writing group deliberations and broader stakeholder input; and (2) usability evaluation through pilot testing with non-expert users, followed by final revisions. Across development phases, checklist elements were refined to (1) prioritize reporting items that most strongly influence outcome interpretation, (2) reduce redundancy across items, and (3) improve decision clarity for commonly misunderstood constructs (e.g., minimum fidelity signals, implementation outcomes versus effectiveness outcomes, and outcomes over time (proximal, intermediate, and distal) [2,36,47,48,49].

### Phase I: Need for Reporting Guidelines and Establishment of Steering Committee/Writing Group

The steering committee is a subcommittee of the International Competency-based Health Professions Educators Collaborative (ICBHPE), established in 2009. Approximately 90 scholars, leaders, implementers, and evaluators of CBHPE from six continents and diverse professions and backgrounds comprise ICBHPE. The aim of ICBHPE is to advance CBHPE through scholarship, implementation support, and international collaboration. The Collaborative has an extensive history of achieving these goals across activities such as journal supplements, world summits, and blog hosting.

In 2024, ICBHPE initiated a consensus process to prioritize emerging needs in the field. Consortium members first brainstormed a broad list of candidate issues and then voted to identify priority topics. Each of the 18 selected topics was then further elaborated by a small working group and approved by the consortium to be discussed at a formal consensus conference, hosted by Stanford University in February 2025. Over 50 participants attended the conference. During the conference, the topics were refined through structured workshops and large-group deliberation, and the view of CBHPE as defined by Van Melle’s Core Components [2] was debated and reaffirmed. The need for a reporting guideline was identified as a top priority.

The Writing Group consisted of 16 members across 4 continents, with 3 serving on the Steering Committee (AC, DR, JF). The writing group/steering committee brings extensive experience in CBHPE, assessment, and program evaluation across multiple health professions, including medicine, pharmacy, and veterinary medicine, and across undergraduate, graduate, and continuing professional education. Collectively, the authors hold leadership roles in academic institutions, accreditation-related activities, assessment and evaluation units, national and international medical and health professions education organizations, and editorial positions. Several authors have contributed to the conceptualization, implementation, evaluation, and scholarly study of CBME/CBHPE, including the development of core components, outcomes frameworks, and prior guidance on evaluation and reporting. These experiences shape our perspective and represent both a strength and a potential source of bias. As scholars and educators who view CBHPE as a promising and evolving response to limitations of traditional training models, we approach reporting with an explicit commitment to theory-informed design, transparency, and cumulative learning. At the same time, we recognize that our familiarity with CBHPE concepts, language, and infrastructure may influence assumptions about what should be reported or how reporting standards are interpreted. To mitigate this, we intentionally grounded these reporting guidelines in established implementation science, program theory, and evaluation frameworks; engaged in iterative, multidisciplinary dialogue across professions and international contexts; and focused on reporting elements intended to improve interpretability regardless of local models, stages of implementation, or outcomes observed.

### Phase II. Drafting and Structured Discussions

During the 2025 Stanford conference, potential elements and frameworks for reporting guidelines were discussed in a 1.5-hour workshop. Post-conference ideas and notes were disseminated, and two structured virtual discussions were held to explore potential content for reporting guidelines. Following these virtual discussions, an outline was disseminated to elaborate the writing and consensus process and list reporting guideline elements, ensuring alignment with van Melle’s CBHPE core components [2] and program-theory constructs (e.g., outcomes, indicators, fidelity, context, adaptations, and equity considerations) [31]. The methodology for creating the guidelines was established through a consensus process [43,44,45,46,50].

Our author group focused on a recurring need identified through these discussions: greater clarity regarding the assumptions, mechanisms, and intended outcomes of contemporary CBHPE. We sought to translate the existing CBHPE literature and collective experience into practical guidance that could support program design, implementation, and evaluation. This produced a Theory of Change paper [31] that is part of the special series of articles in which this current paper is included [16,17,51,52,53,54,55,56,57,58,59] and the Reporting Guidelines contained in this present paper. The CBHPE Outcomes Theory of Change [31] was used to inform the Reporting Guidelines. In brief, the CBHPE Outcomes Theory of Change [31] is a conceptual framework describing how CBHPE initiatives are expected to produce implementation outputs and outcomes over time by situating CBHPE as a theory-informed program intervention in which core CBHPE components [2], stakeholder behaviors, and context interact to produce proximal outputs and longer-term outcomes. It is based on the definition of theory by Varpio [60] and is built on program theory as described by Funnell and Rogers [41]. Together, the Theory of Change and the Reporting Guidelines are complementary in nature. The former articulates the causal logic and expected pathways of CBHPE. At the same time, the Reporting Guidelines were designed to operationalize this logic into standardized reporting practices to improve transparency, interpretability, and comparability across CBHPE studies.

Post-conference, writing groups further developed the products. They presented them back to the consortium for member-checking with revisions informed by feedback to improve clarity, shared meaning of terminology, and usability across countries and professions.

### Phases III and IV. Guideline Development, EQUATOR Registration, and Consensus-Building

Based on feedback, members of the Steering Committee (AC, DR) prepared another draft of the reporting guidelines, which were then circulated to all authors for iterative refinement. Two town hall meetings with the ICBHPE collaborative were conducted to further refine, build consensus, and finalize the guidelines. To support transparency and dissemination, the development team registered the reporting guidelines with the EQUATOR Network reporting guideline registry [42].

Town halls were held virtually in January 2026 with ICBHPE members. These served as structured mechanisms to gather feedback from the ICBHPE. Disseminating this to the ICBHPE membership enabled individuals representing the Global South and other health professions to provide feedback. Discussion prompts were used to solicit input on the clarity of checklist items, the perceived importance of each reporting element, the feasibility of reporting in real-world studies, and areas requiring additional guidance (e.g., definitions, examples, and decision rules). This process of incorporating broad stakeholder engagement aligned with recommended steps for developing health research reporting guidelines, including consultation with relevant end users to strengthen acceptability and real-world applicability [44].

Following each town hall, the members of the Steering Committee (AC, DR) synthesized stakeholder feedback and revised checklist items through iterative rounds of editing. Revisions informed by town halls included consolidating redundant checklist prompts, standardizing terminology across manuscript sections, and adding brief examples to guide interpretation of high-impact items (e.g., fidelity signals, adaptation reporting, and proximal versus distal outcome classification). These refinements were intended to reduce the reporting burden while preserving the elements most essential to the interpretability and transferability of CBHPE outcome findings. This iterative approach followed consensus-method guidance emphasizing transparent synthesis and structured refinement between feedback cycles [43]. Throughout the entire process, the Writing Group members provided feedback on the revisions.

A revised version of the checklist and accompanying explanations was circulated to the writing team members for final review. This stage focused on confirming completeness and clarity, identifying remaining ambiguities, and ensuring that the guidance was applicable across professions and across the educational continuum. Finalization decisions were made through group consensus following structured discussion of any unresolved issues, consistent with common approaches described in consensus methodology guidance [43,44,45,46,50]. The original list of items and the list prior to pilot testing can be found in Appendix II.

#### Pilot Testing and External Review

To strengthen usability and reduce interpretive variability, a pilot evaluation was conducted. Three authors (AC, DR, JF) applied the draft reporting checklist to one or more published CBHPE or CBE outcomes studies. They also provided structured feedback on item clarity, perceived redundancy, feasibility, and areas requiring additional explanation or examples. Pilot testing aligns with guidance for reporting guideline development that emphasizes ensuring end-user usability and feasibility before final dissemination. This pilot stage was also intended to identify elements that could inadvertently introduce reporting burden without improving interpretability. For example, through the pilot, it was identified that the checklist should be structured to identify elements core to all CBHPE studies and to differentiate elements applicable to different study designs or types of CBHPE evaluation.

A Town Hall was held with non-expert users, defined as individuals engaged in health professions education research or curriculum work who were not members of the writing group and lacked specialized expertise in CBHPE outcomes modeling. They were asked to provide feedback on item clarity, perceived redundancy, feasibility, and areas requiring additional explanation or examples. Changes noted by the non-expert users are listed in Appendix II.

#### Final Revisions and Checklist Stabilization

Feedback was reviewed by the author group and incorporated through final revisions. Finalization focused on improving language clarity, standardizing terminology, and ensuring alignment between checklist items and the underlying CBHPE Outcomes Theory of Change [31]. After achieving consensus through revisions, the final checklist was completed (see Table 1). The finalized guidelines were also presented and shared in full with the ICBHPE during a monthly virtual meeting, inviting any final feedback and comments from members. No changes were made from that presentation and discussion. Of note, one change was made during the peer review process, as detailed in Appendix II.

**Table 1:** Reporting Guidelines Checklist.


SECTION	CHECKLIST ITEM	BRIEF DESCRIPTOR	CORE FOR ALL STUDIES	CORE FOR DESCRIPTIVE/EXPLORATORY (INCL. QUALITATIVE)/THEORY-GENERATING STUDIES	CORE FOR IMPLEMENTATION/INTERVENTION STUDIES	REPORTED (YES, NO, PARTIAL, N/A)	PAGE/LINE

**TITLE AND ABSTRACT**	1. Identification	Identify the work as a competency-based health professions education (CBHPE or the discipline-appropriate acronym, such as CBME, CBPE, CBVE, etc.) or initiative in the title AND abstract, including learner level and setting.	✓				

	2. Study Type	Specify whether the work is an evaluation of a CBHPE study and report the study design (e.g., mixed methods, observational, quasi-experimental, qualitative).	✓				

	3. CBHPE Core Components [2]	Identify which CBHPE core components were addressed and indicate the degree of implementation (planned/partial/full).	✓				

	4. Context and Scope	Describe the CBHPE setting (single vs multi-site), participant groups, scope (pilot vs program-wide), and duration.	✓				

	5. Study Purpose	State the primary outcome(s)/study purpose related to CBHPE and, if applicable, specify whether outcomes are proximal (in-training), intermediate (transition to practice), or distal (in practice).	✓				

	6. Fidelity and Study Outcomes	Report the CBHPE-related study outcomes.	✓				

	6A. Implementation or Other Intervention Outcomes	Report key implementation elements (e.g., feasibility, reach, adoption) and/or other outcomes (e.g., clinical, educational, social/behavioral) associated with CBHPE.			✓		

	6B. Fidelity Evaluation	Provide a brief fidelity evaluation, i.e., implementation delivered as intended, if a CBHPE implementation study.			✓		

	6C. Summary of Key Insights	Summarize the CBHPE-related central themes/constructs/relationships identified.		✓			

	7. Conclusions	Provide CBHPE-related conclusions aligned with the evidence.	✓				

	8. Key Words	Utilize “Competency-Based Health Professions Education” (CBHPE) as a key word. Supplement with any discipline-specific iterations of CBE as appropriate.	✓				

**INTRODUCTION**	9. Problem/Rationale	Position the study in the context of the current CBHPE literature. Describe the problem, hook, gap addressed, and explain why a CBHPE approach was selected.	✓				

	10. Conceptual Framing	Anchor the innovation to CBHPE core components and state which components are emphasized.	✓				

	11. Objectives	Clearly state the primary and secondary objectives of the study and indicate whether the focus is CBHPE effectiveness, implementation, or outcomes evaluation (e.g., readiness to implement CBHPE, fidelity of implementation, assessment program validity, educational outcomes, clinical outcomes).	✓				

	12. Definitions	Reference operational definitions for key CBHPE terms. If possible, provide definitions for key and any undefined terms used in the manuscript as a glossary/appendix.	✓				

**METHODS**	13. Theoretical Framework & Role in Study	**Describe the theoretical framework and its function in the study**. Specify whether it is used to: a) **justify/design the CBHPE intervention** (e.g., theory of change, program theory, implementation framework, assumptions/mechanisms), and/or b) **guide the inquiry/analysis/interpretation** (e.g., conceptual lens, epistemological stance, analytic framework).	✓				

	14. Context and Setting	Describe the institutional, educational, regulatory, and/or clinical context, including the pre-CBHPE baseline state.	✓				

	15. Learner Level	Specify learner stage and number of learners.	✓				

	16. Scope of Implementation	Describe the scope of CBHPE implementation (pilot vs program-wide; duration/time window).			✓		

	17. Stakeholder Role Changes	Describe intended changes in behaviors and/or roles of stakeholders associated with CBHPE and how these were developed, and how uptake was assessed.			✓		

	17A. Learners	Describe intended changes in behaviors and/or roles of learners associated with CBHPE and how these were developed, and how uptake was assessed.			✓		

	17B. Others	Describe intended changes in behaviors and/or roles of faculty, preceptors, and administrators associated with CBHPE, how these were developed, and how uptake was assessed.			✓		

	18. Resources and Constraints	Report resource context relevant to CBHPE feasibility (e.g. regulatory/accreditation drivers, faculty availability, infrastructure, time, cost).			✓		

	19. CBHPE Core Components Mapping	Map intervention elements to CBHPE core components.			✓		

	19.1. Outcome Competencies	Describe how learner competencies were selected, defined, aligned, and made transparent.			✓		

	19.2. Sequenced Progression	Describe the developmental progression model, EPAs/milestones or levels, decision points, calibration practices, and/or decision-makers (ex: competency committee).			✓		

	19.3. Tailored Learning	Describe mechanisms for individualized learning, coaching, pacing flexibility, and learner agency.			✓		

	19.4. Instructional Methods	Describe teaching and coaching strategies intentionally aligned with competency development.			✓		

	19.5. Programmatic Assessment	Describe the assessment system, including tools, frequency, data aggregation, entrustment decisions, and assessor development.			✓		

	20. Implementation Strategies	Describe implementation strategies, governance, training, stakeholder engagement, and rollout process.			✓		

	21. Fidelity Monitoring	Specify how fidelity was measured/assessed (implemented as intended) of the CBHPE implementation, such as outputs, uptake/adoption, monitoring methods, and planned vs enacted adaptations.			✓		

	22. Evaluation Design	Describe the CBHPE evaluation design (e.g., rapid evaluation, realist evaluation, logic model, quantitative, qualitative, mixed methods), data sources, measures, analytic approach, and stakeholder involvement.	✓				

	23. Evaluation Focus	Specify whether the CBHPE evaluation focuses on implementation, educational outcomes, or other elements (e.g., Readiness to Implement CBHPE, Fidelity of Implementation, Validity of the Program of Assessment, Educational Outcomes, Clinical Outcomes).	✓				

	24. Future Evaluation (Recommended not Required)	For early-phase CBHPE innovations, describe the planned evaluation approach and CQI integration.			✓		

**RESULTS**	25. Fidelity Results	Report CBHPE fidelity (implemented as intended), such as adherence, level of integration, quality, engagement, and differentiation from baseline.			✓		

	26. Outcome Timing	Report CBHPE-related outcomes by time, whether proximal (in-training), intermediate (transition to practice), or distal (in practice).			✓		

	27. Outcomes	Report CBHPE-related outcomes appropriately based on study design. If quantitative, denominators and measurement methods should be included.	✓				

	28. Monitoring Indicators	Report CBHPE indicators used to monitor outputs and guide continuous quality improvement.			✓		

	29. Adaptations	Describe CBHPE adaptations, timing, rationale, and impact on core versus adaptable elements.			✓		

	30. Contextual Variation	Report CBHPE implementation elements stratified by site or resource context, where applicable.			✓		

	31. Impact on Stakeholders	Report CBHPE impact on stakeholders and any relevant characteristics.			✓		

	31A. Learners	Report CBHPE impact on learners and any relevant learner characteristics. Examples include entrustment decisions (progression, time variability, and remediation), satisfaction, motivation/engagement.			✓		

	31B. Other Stakeholders	Report CBHPE impact on other stakeholders (e.g., faculty, clinician educators, coaches, administrators) and any relevant stakeholder characteristics.			✓		

	32. Assessment Synthesis	Describe how and by whom CBHPE assessment data were aggregated and interpreted longitudinally.			✓		

	33. Program Outcomes	Report CBHPE program-level outcomes, e.g., related to progression, changes in assessments, and decision outcomes/processes.			✓		

	34. Barriers and Unintended Consequences	Report CBHPE challenges, unintended effects, and mitigation strategies.	✓				

	35. Equity by Design	Describe equity-oriented design features embedded within the CBHPE model. (If not included, use N/A.)			✓		

**DISCUSSION**	36. Interpretation	Ensure that the discussion interprets the findings in a manner consistent with the evidence generated, considering the CBHPE core components, theoretical frameworks, and existing CBHPE literature.	✓				

	37. Mechanisms	Explain plausible mechanisms of CBHPE impact using data, such as implementation, fidelity, adaptation, and context.	✓				

	38. Barriers and Facilitators	Identify CBHPE barriers and facilitators. If possible, use an established implementation science framework (e.g., CFIR) or theory.	✓				

	39. Transferability and Scale	Discuss core versus adaptable elements of CBHPE, resource requirements, and implications for transferability.	✓				

	40. Limitations	Discuss CBHPE design, implementation, and external validity limitations.	✓				

**OTHER**	41. Reflexivity Statement [Recommended, not required]	Add a statement identifying how the research team’s background, etc., may impact the CBHPE study findings and work.	✓				

**CONCLUSION**	42. Summary of Insights		✓				

	42A. Summary of Intervention/Implementation Insights	Summarize CBHPE components implemented, key outcomes, implementation adoption, and lessons learned.			✓		

	42B. Summary of Qualitative Insights	Summarize the CBHPE-related central themes/constructs/relationships identified. If theory-generating, then state the proposed conceptual model/mechanisms (or what is newly clarified) and what remains uncertain.		✓			

	43. Practice Implications	Describe implications for CBHPE and potential actionable elements for adoption, including minimum requirements and conditions.			✓		

	44. Future Research	Recommend next steps for CBHPE, i.e., evaluation and evidence building. Describe how this study advances CBHPE knowledge.	✓				


An expansion of the reporting guidelines, with information on how to complete each checklist item, can be found in Table 2. A “not applicable” column with comments has been created, as some study designs may not be able to report all elements. However, these items are comprehensive and worth considering when reporting CBHPE research. Additional reporting guidelines should be utilized that align with your research methodology to ensure reporting. These guidelines are intended to complement existing guidance with specific elements to address surrounding CBHPE. **Scope and Use of this Checklist** This checklist specifies CBHPE-specific reporting elements to address when a study examines, implements, evaluates, or theorizes about competency-based health professions education. It is intended to be used in addition to, not instead of, established reporting guidelines appropriate to the study’s methodology [42], such as qualitative studies (e.g., SRQR, COREQ), observational quantitative studies (e.g., STROBE), implementation studies (e.g., StaRI), and quality improvement studies (e.g., SQUIRE). Authors should first ensure methodological completeness using the relevant guideline(s) and then apply this checklist to report CBHPE-specific elements. **Instructions for Completing the Checklist** Before scoring, identify the study’s primary CBHPE purpose/study type (e.g., descriptive/theory-generating vs implementation/intervention). For each checklist item, indicate Yes, Partial, No, or Not Applicable (N/A) based on the criteria below. When selecting Partial, No, or N/A, provide a brief note (1–2 sentences) indicating what was missing or why the item was not applicable. If an item is reported, score it (Yes/Partial) even if it is not core; use N/A only when it is not expected and not reported.
**Yes:** Select Yes if the item is explicitly addressed and sufficiently described in the manuscript, such that the relevant CBHPE element is clearly identifiable and enough detail is provided for readers to understand what was done, why, and how it informs interpretation of the findings. **Partial:** Select Partial if the item is mentioned or implied, but key details are missing, unclear, or underdeveloped, or the item is addressed in one section but not integrated across the manuscript where expected. **No:** Select No if the item is core to the study’s CBHPE purpose, but it is not reported anywhere in the manuscript, or the reporting is insufficient to support interpretation, transferability, or reuse. **Not Applicable (N/A):** Select N/A only when the item is not core to the study’s CBHPE study type/purpose and is therefore not expected to be reported. Rule of thumb: if a reasonable reader would need this information to interpret the CBHPE claims being made, it is not N/A.
**Additional Guidance**
Multiple standard reporting guidelines may apply. This checklist assesses CBHPE-specific reporting elements and should be used in addition to appropriate methodological reporting standards. Items marked Partial or No should be considered opportunities for improving transparency, not judgments of study quality. This checklist is intended to support consistent reporting, not to serve as a risk-of-bias or quality appraisal tool. For mixed-methods or hybrid papers addressing multiple CBHPE purposes, complete all items that are core to each purpose addressed. Checklist items can be moved to different paper sections (ex: moving conceptual framework from the Methods to the Introduction), depending on the journal selected and the type of study.

**Table 2:** Glossary Of Terms.


TERM	DEFINITION

Competency-Based Health Professions Education (CBHPE)	Outcomes-oriented approach in which competencies are explicit, progression is developmental (not primarily time-based), learning experiences are aligned to competencies, and assessment supports longitudinal decisions about readiness for practice in health professions education [2].

CBHPE Core Components	Five foundational building blocks used to describe CBHPE as a coherent system (e.g., outcome competencies, sequenced progression, tailored learning experiences, competency-focused instruction, programmatic assessment) [2].

Theory of change	An explicit explanation of how and why an initiative is expected to work, specifying assumptions and mechanisms and linking activities to outputs and outcomes over time [41].

Program theory	The set of testable assumptions and causal propositions that connect the CBHPE design and implementation to expected outputs and outcomes; often operationalized via a logic model and accompanying narrative [61].

Implementation strategy	The methods or actions used to adopt, operationalize, and sustain an initiative in a real setting (e.g., training, coaching infrastructure, workflow redesign) [65].

Implementation outcomes/outputs	Outcomes or early observable indicators that reflect the success/quality of implementation (distinct from educational, practice, or patient outcomes), such as acceptability, adoption, feasibility, fidelity, and sustainability [65,66].

Implementation fidelity	Degree to which an initiative is delivered as intended; fidelity reporting supports interpretation of whether findings reflect the model or implementation failure [66].

Implementation Framework	A structured, theory-informed approach used to organize and explain factors and processes that influence implementation (e.g., determinants, strategies, mechanisms, and outcomes), and/or to guide planning, evaluation, and reporting of how an intervention is adopted, delivered with fidelity, and sustained across contexts [67,68].

Competency committee (e.g., Clinical Competency Committee)	A group that synthesizes longitudinal assessment information to make recommendations about learner progression and readiness and to support defensible, programmatic decisions [69,70,71].

Milestones	A criterion-based developmental framework describing progression within competencies; often used to structure expectations and guide longitudinal assessment and feedback [72,73].

Entrustable professional activities (EPAs)	Units of professional work that can be entrusted once sufficient competence is demonstrated; EPA decisions often imply a supervision level and are supported by aggregated assessment evidence [74].

Entrustment (entrustment decision)	The act of transferring patient care responsibility to a trainee at a specified level of supervision for an EPA. A summative entrustment decision should be supported by multiple observations and contextual considerations [75].

Continuous quality improvement (CQI)	Iterative, data-informed cycles used to monitor and refine processes over time; in CBHPE, CQI often applies to assessment system functioning and implementation performance [76].

Proximal outcomes	Early changes during training that indicate the initiative is functioning as intended (e.g., assessment use, feedback quality, learner development) [2,36,47,49].

Intermediate outcomes	Changes during the transition to practice that reflect the application and consolidation of competencies and movement toward practice readiness [2,36,47,49].

Distal/impact outcomes	Longer-term effects in practice and systems (e.g., sustained performance, workforce effects, patient/population outcomes) [2,36,47,49].

Context	The local conditions (organizational, cultural, resource, policy) that shape implementation feasibility, fidelity, and how mechanisms operate [77].

Barriers/enablers	Factors that impede or facilitate implementation and sustainment (e.g., resources, leadership, culture), often treated as determinants influencing implementation outcomes [77].

Equity	Attention to fairness and inclusion in design, implementation, assessment, and progression so that benefits and opportunities are not systematically distributed unequally [78].


## Description of Reporting Guidelines

The final product of the multi-phase consensus process is a structured reporting checklist intended to improve the transparency, interpretability, and reproducibility of CBHPE outcomes studies, called the CORE-HPE: **C**ompetency-Based **E**ducation **O**utcomes **R**eporting for **H**ealth **P**rofessions **E**ducation (Table 1, Figure 2). The checklist is designed to function as a practical tool for authors, reviewers, and editors to ensure that core features of CBHPE implementation and evaluation are consistently reported, particularly those features that meaningfully shape outcome interpretation across contexts. The reporting guideline is delivered in two complementary formats. Table 1 provides the primary checklist organized by manuscript section to support efficient use during study planning, manuscript preparation, and peer review. Appendix I expands each checklist item with a concise descriptor and rationale to support consistent interpretation across disciplines and educational contexts. Together, these materials are intended to serve as a practical reporting tool while preserving sufficient conceptual specificity to enable readers of CBHPE outcomes studies to judge which CBHPE model was implemented, how it was enacted, and which outcomes can reasonably be interpreted given the implementation maturity and evaluation timeframe. Of note, based on the focus of a given study, some checklist items may or may not apply.

**Figure 2:**
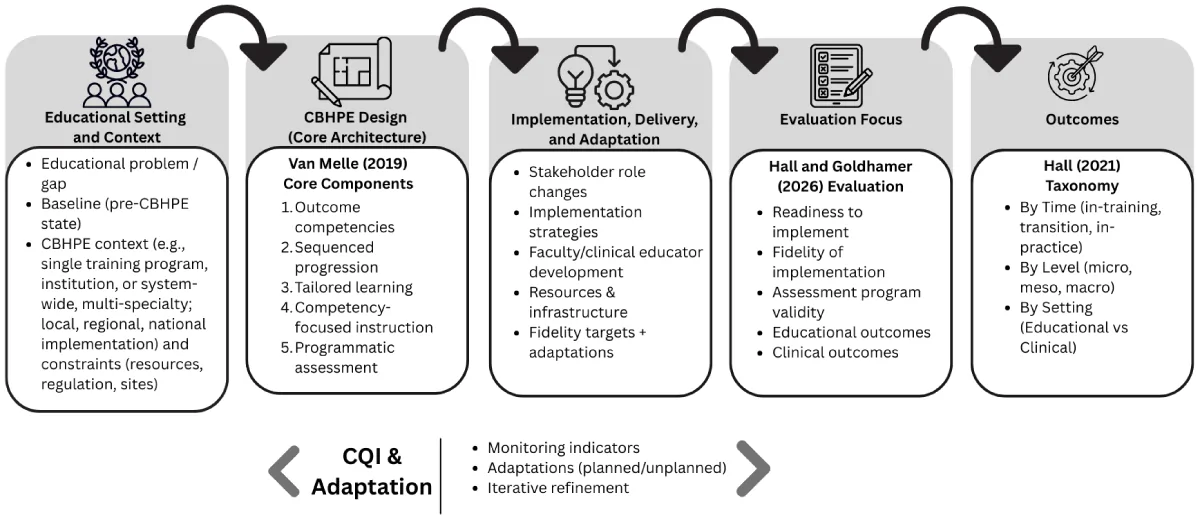
Conceptual Flow Of Cbhpe Design, Implementation, And Evaluation, Illustrating The Theory Of Change And Reporting Domains Addressed In The Cbhpe Reporting Checklist. Key: CBHPE = Competency-Based Health Professions Education. This links to the Theory of Change by providing an explicit explanation of how and why an initiative is expected to work, specifying assumptions and mechanisms, and linking activities to outputs and outcomes over time. Elements linked to the reporting domains are located in the white boxes. Further information can be found in Appendix I, which provides an overview of the details of the Reporting Guidelines.

The checklist was intentionally derived from, and aligned with, the CBHPE Outcomes Theory of Change [31] previously developed by the study team, as noted in Appendix III. Accordingly, the reporting guidelines emphasize reporting elements that strengthen causal interpretability by making the CBHPE system architecture visible, clarifying mechanisms by which outcomes are expected to occur, and documenting conditions that influence implementation success (e.g., fidelity, context, adaptations, and equity considerations). Checklist items map to the outcomes model domains of intervention architecture (core components), program theory (assumptions and mechanisms) [31], implementation conditions (context, enablers/barriers, adaptations, and equity considerations), implementation quality (fidelity), and the nature of the outcomes studied as defined by focus and time from intervention with corresponding indicators [36].

### Organization and Structure of the Checklist

The checklist is organized to mirror how CBHPE outcomes studies are typically communicated in manuscripts and is presented across major sections of a scientific article (e.g., Title/Abstract, Introduction, Methods, Results, Discussion). Within each manuscript section, checklist items prompt reporting of information that directly impacts interpretability, including the CBHPE model being evaluated, implementation conditions, and outcome selection and measurement.

To support practical application, each checklist element includes a brief description and rationale (Appendix I). Where relevant, items encourage explicit differentiation between (1) proximal outputs (early indicators of implementation functioning) and (2) distal outcomes/impacts (educational, clinical, and societal outcomes). This distinction is essential for CBHPE evaluations, given that implementation and learning system maturation often precede measurable changes in downstream outcomes.

### Core Reporting Domains Emphasized in the Guidelines

Across manuscript sections, the checklist (Table 1) highlights several core reporting domains that were consistently identified as essential for meaningful interpretation of CBHPE outcomes. To reduce terminology drift and support coherence across the CBHPE outcomes literature, the reporting guideline domains are organized to align with the CBHPE Outcomes Theory of Change Model [31]. The mapping between checklist items and Theory of Change components is made explicit in Appendix II. As an overview of the reporting guidelines, the core reporting domains are summarized below.

**1. Clear specification of the CBHPE intervention and CBHPE core components**

The checklist prompts authors to describe the CBHPE approach as a coherent system (rather than a single tool or isolated curricular change), including which CBHPE core components are present and how they function together. This includes specifying which elements are considered core versus adaptable, enabling readers to evaluate comparability across settings. For the purposes of reporting, CBHPE core elements are defined as those necessary for the initiative to function as a CBHPE model consistent with Van Melle’s CBHPE core components [2], whereas adaptable elements are locally selected design choices that may vary without altering the fundamental competency-based architecture (e.g., specific tools, assessment frequency, or local governance structures).

**2. Program theory and intended causal logic**

The guidelines emphasize explicit reporting of the CBHPE Theory of Change underlying the CBHPE model, including assumptions, mechanisms of action, and the intended causal pathway(s) linking implementation to outcomes. This program-theory orientation supports interpretation of whether outcomes plausibly reflect the intervention as designed [31,41,61]. As previously noted, the CBHPE Theory of Change was developed first, describing the linkages between implementation and outcomes. This then provided the underpinning for the development of the Reporting Guidelines.

**3. Implementation fidelity and adaptations**

Because CBHPE outcomes are highly sensitive to how consistently a model is enacted, checklist elements encourage reporting of implementation fidelity, including sufficient detail on what was implemented, the nature and extent of engagement, the quality of enactment, and differentiation from prior practices. The checklist also includes reporting of adaptations and evolution of the model over time, acknowledging that iteration is common and often necessary in CBHPE implementation. For studies focused entirely on CBHPE pre-implementation design, curricular implementation and innovation, or other areas that are not primarily assessment-focused, extra attention can be paid to this domain as the following one will not apply.

**4. Assessment infrastructure and progression decision-making**

The checklist emphasizes reporting on assessment design and interpretation processes if these processes are within its focus. The reporting guideline includes how assessment data are used to provide feedback and coaching to aid development, how they are triangulated, how progression decisions are made, and what governance structures support longitudinal assessment (e.g., competency committees or equivalent progression bodies). These elements were prioritized because CBHPE outcomes can be misinterpreted if readers cannot determine how assessment evidence was generated, synthesized, and used for decisions. It is important to note that a given study could focus on formative assessment, summative assessment, both, or neither (if the study focuses on CBHPE curricular implementation and innovation rather than assessment).

**5. Measurement strategy and indicators**

The checklist prompts reporting of outcomes and indicators at multiple levels, including specification of measurement tools, frequency, validity evidence when available, and analytic approaches. In addition, the guidelines encourage reporting of indicators used to monitor system functioning and continuous quality improvement, reflecting the expectation that CBHPE systems rely on ongoing monitoring and refinement.

**6. Equity considerations and bias monitoring**

Given known risks of differential opportunity, bias, and inequitable progression outcomes, the checklist prompts reporting of equity-oriented design features and equity monitoring processes. These elements aim to support responsible interpretation and dissemination of CBHPE outcomes research.

### Intended Use and Scope

These reporting recommendations are intended for studies that evaluate CBHPE outcomes, broadly defined to include outcomes associated with curricula and curricular changes/innovations, learner progression and readiness, assessment and coaching systems, organizational implementation effects, and downstream impacts on practice or patient-oriented outcomes where applicable. The checklist is designed to be used prospectively (during study design and manuscript preparation) and retrospectively (during peer review and appraising the work as a reader of the published product) to support consistent reporting and reduce avoidable ambiguity and insufficient detail in interpreting findings.

Equally important with what these guidelines are, is what they are not. These reporting guidelines are intended to support and enhance, not constrain, CBHPE scholarly output. They are not a prescription but rather a flexible framework designed to enhance clarity, transparency, and interpretability of CBHPE research. We recognize that CBHPE is implemented across diverse contexts and uses a wide range of study designs and methodological approaches. As such, not all elements of these guidelines will be relevant to every study. Authors are encouraged to use the components that align with their work, indicate when items are not applicable, and prioritize reporting that best reflects the purpose, design, and context of their study. Importantly, adaptation is expected. When elements of CBHPE or aspects of study design are modified to fit local needs or constraints, we encourage authors to describe these adaptations explicitly, as they are critical to understanding implementation, context, and interpretation of findings. That is why we included this as a checklist item. We also acknowledge the value of existing and future scholarship that may not explicitly follow these guidelines. High-quality contributions to the field take many forms, and meaningful insights can emerge from a variety of approaches. These guidelines are intended to complement, not replace, diverse scholarship within CBHPE. Ultimately, we invite authors to engage with these guidelines as a resource to support more transparent reporting, enable clearer interpretation of findings, and contribute to a more coherent and cumulative body of CBHPE literature.

The checklist is not intended to serve as a quality appraisal tool for assessing the methodological rigor of CBHPE outcomes studies. Rather, its primary purpose is to improve reporting completeness and interpretability such that readers can understand what was implemented, how it was enacted, the conditions under which it occurred, and how outcomes were assessed and attributed. When applied consistently, the reporting guidelines are expected to strengthen the CBHPE evidence base by enabling more meaningful cross-study comparisons and more trustworthy synthesis of outcomes across settings, institutions, specialties, health professions, and countries. Authors are encouraged to cite this guideline when reporting CBHPE outcomes studies and to submit a completed checklist as an appendix or supplementary file where journal processes permit.

Box 1 provides a role-based roadmap for applying the guidelines across these stages and reinforces a central interpretive principle: CBHPE outcomes are most meaningful when the CBHPE system is sufficiently visible; that is, what was implemented, how it functioned in practice, and which outcomes are reasonable to interpret given implementation maturity and timeframe are clear and sufficiently detailed [44].

**Box 1. Role-Based Application of the Cbhpe Outcomes Reporting Guidelines**

**Figure:**
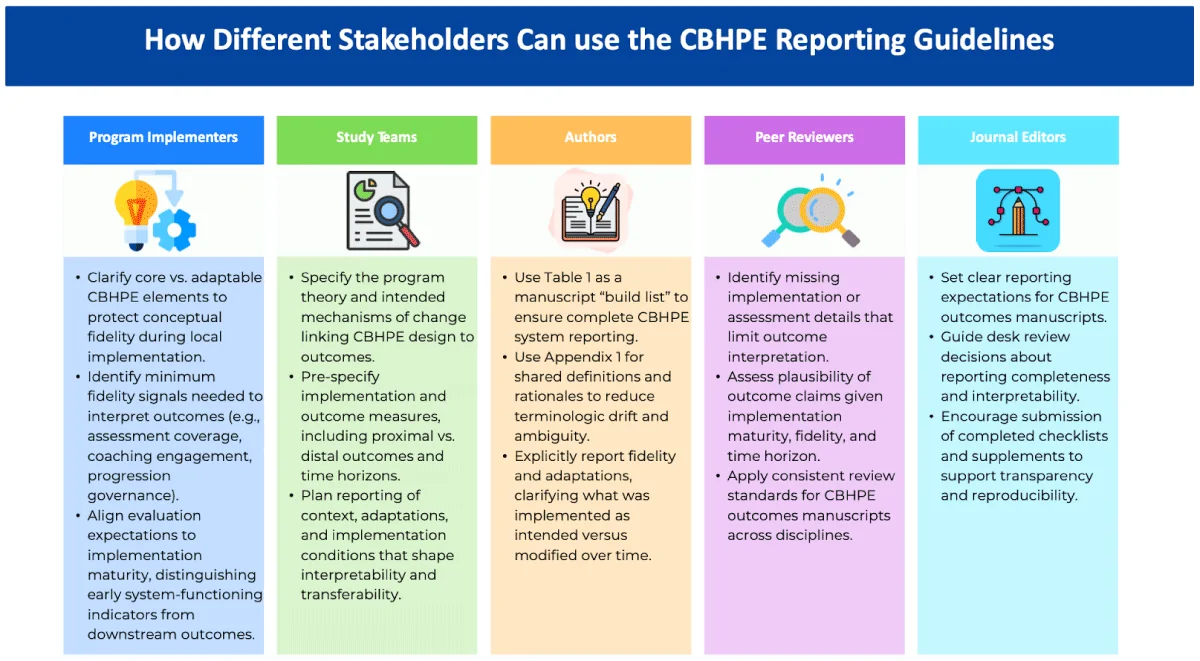

The figure above provides a role-based entry point for applying the CBHPE Outcomes Reporting Guidelines and accompanying checklist (Table 1) to support transparent, interpretable, and transferable reporting of CBHPE outcomes studies. The central premise is that outcomes in CBHPE cannot be meaningfully interpreted when the CBHPE intervention is described only in terms of isolated tools or components (e.g., EPAs, milestones, assessment instruments), rather than as an integrated educational system. For outcomes to be interpretable, readers must be able to determine (1) what CBHPE model was implemented, (2) how it functioned in practice, and (3) which outcomes are reasonable to interpret given implementation maturity and evaluation timeframe.

Accordingly, the reporting guidelines and checklist are intended for use at multiple points across the CBHPE lifecycle. During implementation planning and evaluation design, Table 1 can be used to clarify core versus adaptable elements, align measures with program theory, and specify appropriate proximal and distal outcomes. During manuscript preparation, the checklist supports complete and consistent reporting of CBHPE system architecture, implementation conditions, and outcomes, with Appendix I providing item-level definitions and rationales to support consistent interpretation. During peer review and editorial decision-making, the checklist can be used to standardize expectations for reporting completeness and to identify missing information that limits interpretability, transferability, and synthesis.

Readers should use this figure as a navigation aid to identify their role, then apply the relevant sections of the checklist in Table 1, consulting Appendix I for additional guidance on reporting high-impact domains, including implementation fidelity, adaptations, governance of progression decisions, contextual influences, and equity considerations.

A practical way to apply the checklist (Table 1) is to use it in three steps:

1. Specify the CBHPE model being evaluated, including which core components are present and what is considered core versus adaptable (i.e., adaptable elements are locally selected design choices that may vary without changing the competency-based architecture, such as specific tools, assessment frequency, or local governance structures) [2].
2. Describe the theoretical framework or program theory (assumptions and mechanisms) linking implementation to the outcomes under evaluation [32].
3. Report the evaluation strategy, including how outcomes were selected, the level and timeline at which they were expected to manifest, and how progression decision-making was supported by assessment governance [36].

## Discussion

Created through a consensus-building process [43,44,45,46,50], this article provides a structured set of reporting recommendations for CBHPE outcomes studies that are explicitly aligned with program theory [41] and the system-level architecture required for implementation. The CORE-HPE checklist emphasizes reporting elements that most directly shape interpretability and transferability across programs, including clear specification of CBHPE core components [2], the CBHPE Theory of Change [31], implementation fidelity and adaptations, assessment governance for progression decisions, and outcome selection across time horizons. This work builds directly on the accompanying CBHPE Outcomes Theory of Change [31], offering a practical mechanism to operationalize that model in scholarly reporting and to reduce ambiguity in how CBHPE outcomes are interpreted and synthesized. In doing so, the guidelines may facilitate more transparent, theory-of-change-aligned reporting that enables readers to assess implementation fidelity, interpret outcomes appropriately, and compare findings across contexts. This alignment also helps distinguish between variation due to implementation, context, or underlying theory, strengthening the validity and interpretability of CBHPE outcomes research. Further, adaptation is expected within CBHPE, which does not inherently imply lower fidelity; thus, the guidelines encourage reporting of such adaptations.

These recommendations are intended to address the needs of reporting CBHPE implementation and outcomes, where interpretability depends not only on the competency framework itself, but on how the broader CBHPE infrastructure (workplace learning, coaching, assessment, and progression) operates over time. For example, an outcomes evaluation of a CBHPE initiative introducing EPAs and a competency committee for progression decisions should report not only distal learner performance outcomes, but also implementation signals that establish interpretability (e.g., assessor participation and observation volume, feasibility and adoption across sites, and a minimum fidelity indicator demonstrating that committee review and progression decisions were implemented as intended). Early findings such as improved narrative feedback quality or increased assessment sampling are appropriately interpreted as proximal indicators of system functioning. In contrast, claims about readiness for independent practice or downstream clinical impact require longer follow-up and stronger linkage to sustained programmatic assessment processes [40].

The CORE-HPE reporting guidelines address these challenges by making the CBHPE system visible and by encouraging authors to report essential features needed to understand causal plausibility and contextual dependence. This approach supports interpretability by reducing ambiguity and encouraging a realist lens [62] that seeks to understand what works, for whom, under what circumstances, and why. This is achieved through seeking to understand context-mechanism-outcome relationships. This approach also supports transferability by allowing readers to assess whether the conditions and architecture of an intervention align with their own context. Importantly, the guidelines emphasize distinguishing between proximal outputs (e.g., assessment system functionality, coaching engagement, decision governance) and distal outcomes and impacts (e.g., readiness for practice, patient or system outcomes). This structure mirrors the multi-level outcomes framing proposed in the CBME outcomes taxonomy and supports more appropriate claims about what an outcomes study can and cannot conclude based on the timeframe, implementation maturity, and strength of measurement [36]. As CBHPE outcomes research has expanded, the absence of shared reporting expectations has hindered peer review, replication, and cross-study comparison. This guideline provides a practical, theory-informed framework to support consistent reporting and improve the interpretability, transferability, and scholarly utility of CBHPE outcomes studies.

It is important to note that while the core components of CBHPE [2] are intentionally defined to allow contextual adaptation, they are not simply checklists. For each component, there is a range of uses that can appropriately be considered competency-based, as well as approaches that, despite using similar language or tools, do not substantively achieve the component. Distinguishing warranted adaptation from insufficient implementation is, therefore, a central fidelity challenge in CBHPE and has direct implications for interpreting outcomes.

A major strength of this work is the use of a multi-phase, iterative consensus-development process, informed by established approaches and submitted to EQUATOR, followed by iterative revisions by the ICBHPE and the Writing Group members who represent different professions, continents, and experiences. Importantly, the guideline development process incorporated both in-person workshops and virtual structured discussions, ensuring rich, iterative dialogue across contexts. Member checking and repeated rounds of review further strengthened clarity, shared meaning, and usability across countries and professions. Collectively, these features enhance the rigor, credibility, and global relevance of the resulting reporting guidelines.

### Limitations

This guideline should be interpreted in light of several limitations. First, while the checklist was developed using an iterative consensus-development process informed by established guidance, consensus processes inevitably reflect the perspectives and experiences of the contributors and stakeholders involved. Broader testing across additional professional groups and implementation contexts may identify opportunities to refine terminology, clarify feasibility expectations, or provide additional examples. The Collaborative and the author team for this paper have a heavy Global North representation, with less representation from the Global South. Therefore, while our Global North experiences inform our perspective, they also risk a Global North bias and an inability to fully understand how our work is best conceptualized for meaningful applicability to Global South contexts. This underscores that the checklist should continue to be refined over time, and that programs should be encouraged to report adaptations (as noted in the checklist) to address these limitations. Additionally, we welcome other perspectives, including those who have previously written about the limitations of CBHPE, to continue refining this checklist.

Second, the checklist is intended to improve reporting completeness and interpretability, but it is not designed as a tool to judge study quality or risk of bias. Reporting guidelines support transparency and reproducibility but do not guarantee methodological rigor; rather, they enable readers to evaluate rigor more accurately [44].

Third, the reporting burden remains a legitimate concern, particularly for programs with limited infrastructure or support staff. To mitigate burden, the checklist prioritizes elements that most strongly affect interpretation; however, journal word limits may require authors to use appendices or supplementary materials to document key implementation features fully. Representative examples of how review articles, manuscripts describing innovative CBHPE models, and CBHPE program implementation and evaluations use appendices and supplemental features are cited here for reference [19,63,64]. Further, as flexible guidelines rather than prescriptive standards, these guidelines may not be fully applicable to all studies. Their use should be adapted to context and design, with transparent reporting of modifications and acknowledgment of non-applicable elements.

### Future Directions

Future work should include broad piloting of the CORE-HPE checklist across disciplines and across stages of the educational continuum to assess feasibility, usability, and contribution to reporting completeness. In addition, evaluation studies could examine whether adoption of the checklist improves interpretability and consistency of reporting over time, including the ability to synthesize findings across programs, thereby allowing necessary revisions to the guidelines. To support uptake, an Explanation and Elaboration (E&E) companion document with concrete examples and preferred reporting language would likely improve usability, particularly for authors who are new to CBHPE scholarship or evaluating hybrid or evolving models. Finally, dissemination strategies that include journal endorsement and alignment with broader reporting guideline infrastructures (e.g., EQUATOR [42]) could support standardization and normalization of reporting expectations in CBHPE outcomes research.

## Conclusion

CBHPE outcomes research is essential for guiding educational transformation. Yet, its value is constrained when manuscripts do not clearly describe what was implemented, how it functioned, and under what conditions outcomes were observed. The CORE-HPE reporting recommendations presented in this paper provide a structured, CBHPE-specific checklist aligned with a CBHPE Outcomes Theory of Change [31] to strengthen transparency, interpretability, and transferability across contexts. By supporting consistent reporting of the CBHPE core components, program theory, implementation fidelity and adaptations, assessment and progression governance, outcomes and indicators, and equity considerations, these guidelines offer a practical tool for authors, reviewers, and editors. Authors could consider consulting this CORE-HPE guideline during the planning phases to ensure completeness of the design. Widespread adoption of these recommendations has the potential to improve comparability across studies, strengthen evidence synthesis, and accelerate learning about what works (and does not work) in CBHPE, where, for whom, under what circumstances, and why.

## Disclaimer

The opinions expressed in this article are those of the authors and do not necessarily reflect American Medical Association policy or any member of the ChangeMedEd consortium or other Federal or Governmental Agencies.

## Additional File

The additional file for this article can be found as follows:

10.5334/pme.2554.s1 Appendices. Appendix I to III.
