# Supplementary material for: CORE-HPE Reporting Guidelines: Standardizing Reporting for Competency-Based Health Professions Education Outcomes Research
Source: Perspect Med Educ. 2026 Sep 29;15(1):894–915. doi: 10.5334/pme.2554 (PMC13629552; doi:10.5334/pme.2554)
Supplement: Supplementary file 1 [file pme-15-1-2554-s1.pdf]

APPENDIX I. REPORTING GUIDELINES DESCRIPTORS AND RATIONALE

Element	Description of Element	Rationale for Inclusion
**TITLE AND ABSTRACT**		
**1 and 2. IDENTIFICATION and STUDY TYPE:** Identify as a CBHPE initiative and/or evaluation	• In the title and abstract, identify the work as a CBHPE curricular innovation and specify whether it is an evaluation study. Use the most appropriate term for CBE and the paper context, whether broad (i.e., CBHPE) or discipline-specific (ex: CBME, CBPE, CBVE).<br>• Include the design, e.g., mixed methods, quasi-experimental, observational, qualitative.<br>• Include the learner level and setting in brief (program, specialty, country).	Titles and abstracts may be read only in part and must contain enough information for readers to judge relevance and (at a high level) validity/applicability; reporting guidance for abstracts emphasizes clear identification of what was done and the study design.
**3. CBHPE CORE COMPONENTS:** Core CBHPE components addressed	• Include a brief mapping of which Van Melle core component(s) were central to the innovation (e.g., outcomes competencies, sequenced progression, tailored learning, competency-focused instruction, programmatic assessment) [1]<br>• If feasible in one phrase, note degree of implementation (planned/partial/full).<br>• For reporting purposes, core elements are those required for the initiative to function as a CBHPE model consistent with Van Melle's core components. In contrast, adaptable elements are locally selected design choices that may vary without changing the competency-based architecture (e.g., tools, frequency, governance details).	CBHPE is used inconsistently; explicitly naming which core components were implemented improves conceptual fidelity and helps readers interpret outcomes in relation to what version of CBHPE was actually enacted.
**4. CONTEXT AND SCOPE:** Context, participants, and scope	Briefly state setting/context (single vs multi-site), participant groups (learners; faculty/clinician educators; administrators, if relevant), and scope (pilot vs program-wide; duration/time window).	CBHPE interventions are context-sensitive; minimal context + scope in the abstract improves interpretability and transferability judgments before a reader reaches the full text.
**5. STUDY PURPOSE:** Outcomes and time horizon	• Name the primary outcome(s) or study purpose related to CBHPE.<br>• Specify whether they are proximal (in-training), intermediate (transition to practice), or distal (in practice) [2]; provide the follow-up window (e.g., one term, one year).	Complex interventions can show different effects over different time scales; stating outcome type and time horizon reduces over-claiming and improves cross-study comparability.
**6A, 6B, and 6C. IMPLEMENTATION/OTHER OUTCOMES, FIDELITY EVALUATION, AND SUMMARY OF KEY INSIGHTS:** Study outcomes and implementation outcomes, including fidelity	**If an implementation/intervention study:**<br>• Report key CBHPE implementation outcomes (e.g., feasibility/reach/adoption) if an implementation/intervention study.<br>• Report other CBHPE outcomes as applicable to your study design and purpose, e.g., clinical outcomes, educational outcomes, social/behavioral.<br>• Provide a brief fidelity signal (e.g., whether the intended CBHPE components were delivered as planned; major deviations/adaptations if pivotal) [3, 4].<br>**If a descriptive/exploratory/theory-generating study:**<br>• Summarize your central themes, constructs, relationships, or theories generated in relation to CBHPE.	Implementation outcomes are distinct from effectiveness and are necessary to interpret impact; fidelity is a key threat to interpretation in complex interventions and helps distinguish intervention effects from incomplete/variable delivery.
**7. CONCLUSIONS:** Conclusions and implications	• Provide a CBHPE-related conclusion that matches the evidence and time horizon.<br>• Consider 1 practical implication (e.g., feasibility for further implementation; resource requirements; what should be tested next) (if space allows).	Abstract conclusions frequently drive uptake; structured implications improve usability and reduce mismatch between claims and what was actually evaluated.
**8. KEY WORDS:** Use key words that will make it easier to find implementations of CBHPE	• Utilize "Competency-Based Health Professions Education" (CBHPE) as a key word.<br>• Supplement with any discipline-specific iterations of CBE as appropriate.	Without consistent use of key words, it can be challenging to find studies examining similar topics. Consistent use of terminology and subject headers has been recommended in other disciplines for ease of article retrieval [5].
**INTRODUCTION**		
**9. PROBLEM/RATIONALE:** Problem/gap and why CBHPE is the proposed solution [6-9]	• Specify whether the primary drivers are educational, organizational, and/or societal, and what 'need for change' the innovation is responding to.<br>• Describe the practice/education problem or gap the innovation addresses (what is failing or insufficient, for whom, and why it matters).	Specify whether the primary drivers are educational, organizational, and/or societal, and what 'need for change' the innovation is responding to. Describe the practice/education problem or gap the innovation addresses (what is failing or

	• Briefly justify why a competency-based approach is expected to address the gap (e.g., outcomes focus, developmental progression, learner-centeredness vs time-based assumptions).	insufficient, for whom, and why it matters). Briefly justify why a competency-based approach is expected to address the gap (e.g., outcomes focus, developmental progression, learner-centeredness vs time-based assumptions).
**10. CONCEPTUAL FRAMING:** Using Van Melle's core components [1]	• Briefly describe Van Melle's five core components and state (at a high level) which component(s) the innovation emphasizes.<br>• Keep details for Methods but make the conceptual anchor explicit up front so readers know what approach to CBHPE is being advanced.	CBHPE is frequently discussed inconsistently; using a shared core-components framework improves conceptual fidelity and helps readers interpret later Methods/Results in relation to what was actually implemented.
**11. OBJECTIVES:** CBHPE-related study aims and focus [10]	• Clearly state the primary objective(s) and any secondary objectives.<br>• If applicable, specify whether the report is evaluating effectiveness, implementation (e.g., acceptability/feasibility/adoption) or outcomes evaluation (e.g., readiness to implement CBHPE, fidelity of implementation, assessment program validity, educational outcomes, clinical outcomes), and define the main outcomes at a headline level.	Clear objectives are a basic requirement for interpretable reporting and determine what Methods/Results elements are necessary. For CBHPE, distinguishing effectiveness from implementation aligns with established implementation-outcomes concepts and prevents mismatched conclusions.
**12. DEFINITIONS:** Key terms and definitions used in the report	• Reference operational definitions for key CBHPE terms. Use consistent with other CBHPE studies.<br>• Provide brief operational definitions for essential terms used in the manuscript (e.g., competence, entrustment/levels of supervision if EPAs, programmatic assessment, milestones), especially when terms could be interpreted differently across disciplines or regions.<br>• If possible, provide definitions for key and any undefined terms used in the manuscript as a glossary/appendix. Examples of glossaries are cited here.[11-13]	Shared terminology improves clarity and comparability across CBHPE reports and reduces ambiguity that undermines synthesis and replication. Consistent use of terminology has been recommended in other disciplines for comparability [5].
**METHODS**		
**13. THEORETICAL FRAMEWORK & ROLE IN STUDY:** Frameworks, program theory, and theory of change [14-20]	• Explicit statement of the theoretical framework, program theory, or theory of change guiding the CBHPE intervention, including: (1) key assumptions, (2) mechanisms of action expected to produce change, and (3) the causal pathway linking CBHPE components → stakeholder behaviors → outputs → outcomes.<br>• When feasible, include a simple logic model or diagram and identify which components are core versus adaptable.<br>• Should be used to either justify or design the CBHPE intervention/implementation or guide the inquiry/analysis of the study (e.g., conceptual lens, epistemological stance, analytic framework).<br>• Consider linking to the CBHPE Outcomes Theory of Change [19].	CBHPE outcomes are difficult to interpret without a clear explanation of how the model is expected to work. Reporting the theory of change supports distinction between implementation failure (fidelity/uptake limitations) and theory failure (mechanisms not activated or not plausible). It strengthens interpretability, improves reproducibility, and prevents post-hoc explanation.
**14. CONTEXT AND SETTING:** Institutional/educational context, with rich, detailed descriptions [14, 15, 21, 22]	• Describe the institutional + educational ecosystem: country/region, profession(s), program type (e.g., UME/GME/CPD; degree/residency), institution type (e.g., academic health center, community-based), class/cohort size, admissions/selection context, clinical learning environments (e.g., sites, patient populations), accreditation/regulatory constraints, curricular structure (e.g., credit hours/time-based rules), and baseline pre-CBHPE state (what existed before).<br>• Include timeline and maturity (e.g., pilot vs established).	Context strongly shapes what is feasible and what outcomes mean; poor contextual reporting limits transferability and interpretability. This aligns with broader HPE calls for better reporting quality.
**15. LEARNER LEVEL:** State the learner level [15, 16, 19]<br>**16. SCOPE OF IMPLEMENTATION:** State how broadly CBHPE was implemented [15, 16, 19]	• Specify: learner stage (year/PGY), number of learners affected, whether mandatory/opt-in, inclusion/exclusion, length of exposure, and scope (e.g., single course, longitudinal thread, whole program).<br>• Note whether implementation is single site vs multi-site and whether it spans transitions (e.g., UME→GME).	Without scope/level, readers cannot judge the implementation level, generalizability, or whether outcomes reflect CBME vs. a local micro-innovation. Also supports comparisons across CBME literature conversations where heterogeneity is a known challenge.
**17. STAKEHOLDER ROLE CHANGES:** Key Stakeholder Behavior / Role Changes Required [23-26]	• Description of the intended changes in stakeholder behaviors and roles required for CBHPE to function (e.g., learner self-regulation and help-seeking; faculty/preceptor/clinical educator observation, coaching, entrustment judgments; administrator support for time/resources/governance).	CBHPE is a behavioral and cultural system change, not only a curricular change. Outcomes depend on whether faculty, preceptors, learners, and administrators adopt new behaviors. Reporting stakeholder role shifts improves interpretability of

**17A. LEARNERS**<br>**17B. OTHERS**	• Include how these roles were supported (training, policies, workflow changes) and how uptake was assessed (participation rates, observation/coaching frequency, portfolio documentation, committee decision processes)	results and supports assessment of transferability, scalability, and sustainability across contexts.
**18. RESOURCES AND CONSTRAINTS:** Setting/context-dependent [27-29]	• Report resource-relevant context that affects feasibility of CBHPE (especially curricula and assessment): faculty/preceptor-to-learner ratios, assessor availability, clinical supervision models, patient volumes, geographic dispersion (rural/urban), technology/data infrastructure (portfolio platform, connectivity), administrative support, and time/cost constraints.<br>• If multi-site, describe variation across sites.	Report resource-relevant context that affects feasibility of CBHPE (especially curricula and assessment): faculty/preceptor-to-learner ratios, assessor availability, clinical supervision models, patient volumes, geographic dispersion (rural/urban), technology/data infrastructure (portfolio platform, connectivity), administrative support, and time/cost constraints. If multi-site, describe variation across sites.
**19. CBHPE CORE COMPONENTS MAPPING:** Components addressed mapped to Van Melle 2019 [1]	• Consider providing a simple mapping table: each Van Melle component [1] marked as Primary / Secondary / Not addressed, with 1–2 sentences describing how it appears in the intervention.<br>• (Optional but valuable: report degree of implementation e.g., Initial, partially implemented, fully implemented.)<br>• Address the below elements (19.1-19.5) based on whether the study includes a focus on each particular element.	Van Melle provides a consensus-derived organizing framework; mapping reduces ambiguity in the CBPHE label and helps reviewers assess fidelity to CBPHE principles.
**19.1. OUTCOME COMPETENCIES [1]:** Describe how outcomes were selected and aligned with frameworks related to discipline [18, 26, 30-32]	Report:<br>• (a) competency sources (e.g., CanMEDS/ACGME/CBVE/discipline standards)<br>• (b) how selected (e.g., needs assessment: patient/societal/workforce; stakeholder input)<br>• (c) level of granularity (e.g., competencies vs EPAs)<br>• (d) operational definitions (e.g., observable behaviors)<br>• (e) transparency strategy (e.g., where published; how learners/preceptors access)	Clear, measurable outcomes are foundational to constructive alignment and accountability; your logic model highlights problems when competencies are unclear (e.g., misalignment, stakeholder confusion).
**19.2. SEQUENCED PROGRESSION [1]:** Detail how progression of competencies is structured (e.g., milestones, levels, developmental rubrics) [33-36]	• Describe the developmental model: milestones/levels, how milestones were created/validated, how they map across courses/rotations, and where progression expectations are published (e.g., syllabi/handbooks).<br>• Include decision points (e.g., promotion/ advancement), remediation pathways, and calibration practices (e.g., rater training, shared frames of reference).	CBHPE emphasizes developmental progression over time-based training, so readers need sufficient detail on milestones/levels to understand what progression means and how it was assessed. If milestone structure, performance levels, and rater training are not reported, it becomes difficult to interpret progression outcomes or compare findings across programs.
**19.3. TAILORED LEARNING[1]:** Report on individualized learning plans, flexibility in pacing, or learning pathways [6, 37-40]	Report mechanisms used to individualize learning: ILPs, coaching/mentoring structure (e.g., who, frequency), flexibility in pacing or pathways (e.g., remediation/ acceleration/ enrichment), use of dashboards/ portfolios/ analytics, and how learner agency/reflection is built in (e.g., structured reflections, self-assessment tools).	Tailored learning experiences are a core component of CBHPE, intended to support learner-centered, flexible progression based on individual needs rather than a one-size-fits-all pathway. When authors do not report how learning is tailored (e.g., individualized learning plans, coaching structures, flexible pathways, or mechanisms for using performance data to target experiences), it becomes difficult for readers to judge whether CBHPE principles were enacted and to replicate the approach in other settings.
**19.4. INSTRUCTIONAL METHODS [1]:** Describe teaching strategies tailored to support competency development [41]	Describe teaching practice intentionally aligned to competency development: learning experience types (e.g., workplace-based, simulation, case-based), opportunities for deliberate practice, feedback structures (e.g., timing, who gives it, how captured), scaffolding strategies tied to milestones, and how instruction adapts using performance data. Include what is standardized vs locally adaptable.	Competency-focused instruction is one of Van Melle's five core components of CBHPE and is conceptually distinct from (a) designing learning experiences and (b) assessing performance. It captures the teaching/coaching practices intentionally used to move learners along a developmental trajectory (e.g., feedback structures, coaching models, deliberate practice, scaffolding tied to milestones). Because curricular innovations often collapse teaching practices into generic methods, it becomes unclear whether the intervention truly operationalizes this CBHPE component or relabels existing instruction. Explicit reporting of competency-focused

		instruction also supports replication and synthesis, consistent with the broader purpose of reporting guidance for complex interventions (i.e., describing what was delivered in sufficient detail for others to implement it).
**19.5. PROGRAMMATIC ASSESSMENT [1][[35, 42-45]:**<br>• Explain how data were collected longitudinally across tools and settings.<br>• Include the use of formative and summative assessments.<br>• Describe entrustment, decision-making models, and faculty development.	• Report the assessment system as a program, not a list: assessment blueprint across competencies, tools (WBA, OSCE, exams, portfolios), frequency + stakes, longitudinal data aggregation, narrative data use, triangulation/synthesis process, and who makes progression decisions (e.g., CCC/competency committee [45])<br>• Include entrustment approach (e.g., EPAs/entrustment scales), rules for decisions, and assessor development.	Programmatic assessment is a defining feature of CBHPE because it treats assessment as a system that supports both learning and progression decisions. Core descriptions of programmatic assessment emphasize that many low-stakes, feedback-rich data points are collected over time, intentionally aligned to competencies/EPAs, and then aggregated and synthesized (often with narrative data) to inform holistic judgments and high-stakes decisions by appropriate groups (e.g., competency committees). Without clear reporting on how data points are generated, combined, and used to inform decisions, readers cannot determine whether the approach reflects programmatic assessment principles, interpret outcomes, or replicate the assessment system in another context. When EPAs are used, reporting should also describe entrustment (e.g., supervision/entrustment scales, who makes entrustment judgments, and how entrustment decisions are supported by aggregated evidence), since entrustment is a specific workplace-based judgment that needs transparent operationalization.
**20. IMPLEMENTATION STRATEGIES:** How the change was implemented [22, 46]	Describe how the change was implemented: rollout plan, governance, training, communications, stakeholder engagement (e.g., learners, faculty, preceptors), IT/data infrastructure, resource supports (e.g., time, roles), and key adaptations during rollout.	CBHPE is a complex educational intervention, typically involving coordinated changes across outcomes, teaching practices, workplace learning, and assessment systems. Therefore, learner and program outcomes are often uninterpretable without clear reporting of what was actually implemented, for whom, by whom, and under what conditions (i.e., opening the black box of implementation). Reporting implementation strategies also enables readers to assess key implementation outcomes, such as feasibility, acceptability, adoption, and fidelity, which are essential for explaining variability in results across contexts. Finally, detailed implementation reporting supports replication, adaptation, and scale, consistent with established guidance for reporting complex interventions (e.g., structured reporting via TIDieR [47]).
**21. FIDELITY MONITORING:** Fidelity of CBHPE implementation [4, 48, 49]	• Specify fidelity targets and how monitored: which core components were delivered as intended, adherence/degree to which implemented/exposure, participation rates, rater completion rates, coaching contact frequency, and audit processes.<br>• Report planned vs enacted adaptations (what changed and why?).	Implementation fidelity (the degree to which an innovation is delivered as intended) is a major source of variation that affects the credibility and interpretability of outcomes in complex interventions. Without fidelity reporting, readers cannot distinguish whether results reflect intervention effectiveness or implementation failure (e.g., incomplete delivery, variable dose, inconsistent use across sites/raters), thereby weakening causal interpretation and limiting reproducibility. Fidelity is also frequently underreported in outcome evaluations; classic reviews found that only a minority of studies documented or analyzed implementation integrity [48, 50, 51], even though reduced adherence is often associated with poorer outcomes, therefore, explicit fidelity reporting supports valid

		interpretation, cross-study comparison, and replication/adaptation across settings.
**22. EVALUATION DESIGN, 23. EVALUATION FOCUS, and 24. FUTURE EVALUATION**<br>**For research projects [10, 14, 15, 26, 46]:**<br>• Study design: observational, mixed methods, intervention, etc.<br>• Data collection tools and validation<br>• Stakeholder involvement<br>• Focus of evaluation<br>**For innovations without full study designs yet [10, 15, 52-54]:**<br>• How might it be evaluated going forward?	**Research Projects:**<br>• Report design (e.g., experimental/quasi/observational; mixed methods), theoretical framing, data sources + timing, instrument validity evidence where relevant, analytic approach (e.g., quant/qual integration), and stakeholder involvement.<br>• Separate implementation outcomes from learner/program outcomes (define what success looks like).<br>• Report the focus of the evaluation, e.g., readiness to implement, fidelity of implementation, assessment program validity, educational outcomes, clinical outcomes.<br>**Innovations/Future Evaluation:**<br>• State intended evaluation questions, proposed measures, timeline, data sources (including routine program data), plans for monitoring unintended consequences, and how findings will feed continuous quality improvement (decision-making cadence).	**Research projects:**<br>Clear reporting of the evaluation design is essential because CBHPE interventions are typically complex and context-sensitive; readers need sufficient methodological detail (design, timing, measures, analytic approach) to judge risk of bias, interpret findings, and compare results across programs. In addition to effectiveness outcomes, reporting process/implementation evaluation helps explain why results occurred (or did not) by documenting what was delivered, how it was delivered, likely mechanisms of impact, and contextual influences on variation in outcomes. Using established implementation-outcomes concepts (e.g., acceptability, feasibility, adoption, fidelity, sustainability) strengthens interpretation and supports generalizable learning about implementation. Finally, evaluation reporting should include evidence supporting the quality of measurement and decisions (e.g., validity evidence for instruments and for how scores/judgments are used), consistent with widely used testing/measurement standards.<br>**Innovations:**<br>Clearly delineating the focus of the evaluation enables others to determine the stage of CBHPE implementation and the potential for adaptability to local contexts,<br>CBHPE curricular change is typically a complex intervention that unfolds in phases (development, feasibility/piloting, evaluation, and implementation). Because many innovations are disseminated during early phases (before long-term learner, educational, patient, and/or clinical outcomes can reasonably be observed), explicitly reporting an evaluation focus and plan clarifies where the work sits in the intervention lifecycle and prevents readers from overinterpreting preliminary findings. A stated plan also increases transparency about what outcomes will be measured, when, and how, including planned assessment of implementation outcomes (e.g., acceptability, feasibility, adoption, fidelity, sustainability) that often explain why results vary across contexts. Finally, specifying the planned evaluation approach aligns with established reporting expectations for system and educational improvement work, supporting cumulative learning across settings even when effectiveness outcomes are not yet mature.
**RESULTS**		
**25. FIDELITY RESULTS:**<br>Fidelity of CBHPE implementation [3, 4, 19]	• Report the extent to which the intended CBHPE model was delivered as designed/intended.<br>• At minimum, describe fidelity using established dimensions: (1) adherence to core CBHPE components (what was delivered vs planned; ideally mapped to Van Melle's 5 core components)[1], (2) description of the intervention (how much occurred; frequency/volume of key CBHPE activities), (3) quality of delivery (e.g., quality of coaching/feedback; assessor calibration indicators), (4) participant responsiveness/engagement (learner and assessor participation/uptake), and (5)	Fidelity is essential for interpreting outcomes in complex educational interventions: without it, readers cannot determine whether results reflect the CBHPE model or inconsistent/incomplete delivery. Fidelity frameworks specify key dimensions (adherence, dose, quality, responsiveness, differentiation) that directly affect causal interpretation, cross-study comparison, and replicability.

	differentiation from usual practice (what changed compared with baseline).<br>• Report planned vs delivered, variability across sites/raters/cohorts, and summarize major deviations (with a pointer to the Adaptations item for full details).	
**26. OUTCOME TIMING:** Proximal vs intermediate vs distal outcomes (and time horizon) [2, 14, 15, 19, 46, 55]	• Report CBHPE-related outcomes by time, whether proximal (in-training), intermediate (transition to practice), or distal (in practice).<br>• Specify which outcomes are proximal (near-term signals such as milestone progression, feedback frequency/quality, portfolio engagement, coaching contacts) vs intermediate (e.g., graduation readiness, transition performance) vs distal (e.g., patient/system outcomes), and over what time horizon each was measured.<br>• If distal outcomes aren't available, state this explicitly and justify.	CBHPE interventions function as complex interventions with multiple interacting components and pathways; effects often emerge at different time scales. Distinguishing proximal from distal outcomes reduces over-claiming, clarifies what conclusions are supported by available follow-up, and improves cross-study comparability.
**27. OUTCOMES:** Related to the study design and approach to CBHPE [19, 26, 46, 56-58]	• Report CBHPE-related outcomes appropriately based on study design.<br>• If quantitative, denominators and measurement methods should be included.<br>• Report implementation outcomes with clear denominators and measures (as applicable): acceptability, feasibility (e.g., time, resources, money), appropriateness, level of adoption, fidelity, penetration/reach, sustainability/maintenance.<br>• State the stakeholder(s) measured (learners, faculty, preceptors/clinical educators), method (survey/interviews/admin data), and results.	Implementation outcomes are conceptually distinct from learner/program outcomes and are essential to interpret variation in impact across settings. A taxonomy defines these outcomes and provides a shared language for reporting, synthesis, and replication decisions.
**28. MONITORING INDICATORS:** Indicators/metrics used to monitor outputs and guide continuous quality improvement [14, 19, 52, 59]	Reporting of indicators/metrics used to monitor implementation quality and early outputs (e.g., volume and quality of workplace-based assessments, coaching frequency, portfolio completion, assessment sampling, committee decision rates), including data sources, measurement frequency, and how results informed continuous quality improvement (decision cadence, thresholds/triggers for intervention, governance process).	CBHPE implementation requires monitoring of proximal outputs before distal outcomes can be expected. Reporting indicators demonstrate whether the system is functioning as intended, strengthen interpretability, and support dissemination and replication. Connecting indicators to CQI is essential because CBHPE is dynamic and expected to evolve through iterative refinement.
**29. ADAPTATIONS:** Adaptations/modifications during implementation [19, 58, 60-62]	• Report what changed or evolved during implementation (content, delivery, how much of CBHPE was implemented, personnel roles, setting/format), when it changed, why and how it changed (e.g., feasibility constraints, stakeholder feedback, site variation), and who initiated/approved the change. Indicate whether each modification affected core elements (vs adaptable elements), whether it was planned/proactive or reactive, and whether it was intended to improve fit, fidelity, feasibility, or effectiveness.<br>• Categorize adaptations using something like FRAME [60] and summarize any observed effects of major adaptations on fidelity, reach, or outcomes.	CBHPE innovations commonly evolve during rollout. If adaptations are not reported, replication is compromised, and fidelity claims become ambiguous (it is unclear whether outcomes reflect the original model, a modified model, or site-specific drift). Structured adaptation reporting clarifies tailoring vs erosion of core components and supports responsible transfer to other contexts.
**30. CONTEXTUAL VARIATION:** Implementation performance by resource context [19, 22, 46, 63, 64]	• Report at least feasibility + fidelity + reach/penetration (and resource burden/cost if measured) overall and stratified by relevant site/resource context (e.g., rural vs urban; higher- vs lower-resource sites; different supervision models). Include denominators.<br>• Briefly state the main contextual driver(s) of differences if observed (e.g., assessor availability, workload, connectivity/platform access).	CBHPE implementation is context-dependent; pooled averages can hide site-level variation that drives real-world success or failure. Stratifying key implementation outcomes supports interpretability, informs scalability decisions (including in lower-resource contexts), and enables synthesis across settings.
**31. IMPACT ON STAKEHOLDERS: 31A. LEARNERS and 31B. OTHER STAKEHOLDERS** [19, 44, 65-69]	**Learner**<br>• Report (cohort/aggregate): Learner developmental outcomes aligned to the CBHPE model, with learner/context characteristics needed to interpret results (e.g., stage/level, baseline preparation, context).<br>• Where feasible and appropriate to the study design, report progression-focused evidence such as trajectories/distributions over time (not only averages), progression decisions/rates (e.g., on-track/conditional/remediation/acceleration), time-to-milestone or time-in-stage, and remediation/acceleration supports and outcomes.<br>• If EPAs are used, report entrustment/supervision outcomes over time, the	**Learner**<br>CBHPE claims depend on developmental progression, learner engagement with feedback, and transparent decision-making (including entrustment when EPAs are used). Reporting trajectories, engagement processes, and decision rules allow readers to interpret progression meaningfully, compare programs, and judge whether decisions reflect aggregated evidence rather than isolated observations.<br><br>**Other Stakeholders:**<br>CBHPE implementation depends on the capability,

	supervision/entrustment scale (levels of supervision), and the decision rules/synthesis process used to make entrustment and progression decisions.<br>• When relevant to the intervention, report learner engagement with the developmental process (e.g., self-directed learning/ILP engagement, feedback uptake/coaching engagement) and define instruments/measures used.<br><br>**Other Stakeholders:**<br>• Report (core, cohort/aggregate): Impact on key stakeholders who enable CBHPE (e.g., faculty, preceptors/clinician educators, coaches, administrators), aligned to the CBHPE model, including relevant stakeholder/context characteristics needed to interpret findings (e.g., role, setting, experience/training, expected responsibilities).<br>• Where feasible and appropriate to the study design, report stakeholder impact metrics such as workload/time burden and workflow fit, role changes (e.g., coaching, assessment, competence committee participation), and support/development needs (e.g., training, calibration, coaching support).<br>• When relevant, report indicators of stakeholder engagement and implementation readiness (e.g., acceptability, feasibility perceptions, satisfaction, motivation/engagement, participation rates, intention to sustain).<br>• Consider reporting (system functioning): Measures tied to stakeholder work that illuminate implementation quality (e.g., rater participation and quality, decision-making processes and timeliness, usability of data systems/platforms, administrative burden).	opportunity, and motivation of stakeholders who design, deliver, assess, coach, and govern the program. Reporting the impact on these stakeholders clarifies whether CBHPE was feasible to implement, was enacted with fidelity, and was supported over time within real-world constraints of staffing, time, and infrastructure. Stakeholder impact data also help readers interpret learner outcomes by distinguishing effects attributable to the educational model from those driven by exceptional resources or unsustainable effort, and they surface contextual factors critical for scalability, transferability, and sustainability across settings.
**32. ASSESSMENT SYNTHESIS:** Triangulation, trends, or meaningful pattern [19, 42, 67, 70-73]	• Show how CBHPE-related assessment data were combined and interpreted: trajectories/patterns over time, evidence across tools/settings, narrative synthesis themes, and how multiple data points were aggregated to support judgments (e.g., portfolio synthesis and/or committee outputs).<br>• Include who is involved in the assessment data processes.<br>• Report meaningful patterns rather than single-tool snapshots.	Programmatic assessment is defined by maximizing the learning value of individual data points while aggregating many data points for higher-stakes decisions. Reporting isolated scores does not demonstrate programmatic assessment and limits replication of the synthesis approach.
**33. PROGRAM OUTCOMES:** System-level performance [19, 48, 66, 74]	Report CBHPE outcomes at the program/system level relevant to the innovation scope: progression/graduation decisions, distribution of time to competence (if applicable), assessment volume/burden at program level (e.g., WBAs per learner; committee review cadence/time), changes in assessments, assessor participation rates, decision consistency/dependability indicators (if measured), and CQI actions triggered by the system.	CBHPE is a system change, not only an individual learner intervention. Program-level results demonstrate whether the model functions as intended at scale (in procedures, aggregation, and decision processes) and help other programs assess feasibility and sustainability beyond learner score changes.
**34. BARRIERS AND UNINTENDED CONSEQUENCES:** Challenges, barriers, and unintended consequences [19, 75-77]	• Report CBHPE key challenges/barriers (what, where, who affected) and unintended consequences (e.g., increased burden, rater drift, gaming, learner perception that formative feels summative), plus mitigation steps and lessons learned.<br>• Do not re-list the adaptation details here (reference the Adaptations item above).	CBHPE systems can produce predictable unintended effects that influence feasibility and learning behaviors. Reporting barriers and unintended consequences improve transferability and help interpret whether outcomes reflect the intended mechanisms or implementation-related side effects.
**35. EQUITY BY DESIGN:** Describe how risk for equity differences was evaluated and addressed, particularly in assessment [19, 78-81]	Description of equity-oriented design features embedded within the CBHPE intervention (in addition to outcome monitoring), such as rater training and calibration, bias mitigation strategies, structured progression decision-making processes, safeguards within competency committees, learner support structures, and equity auditing triggers (what prompts review and what actions follow).	Equity is shaped by design and governance decisions across assessment, coaching, and progression. Reporting equity-by-design strengthens fairness and trustworthiness, supports evaluation of differential impact, and promotes responsible dissemination by reducing risk that CBHPE reproduces inequities through new assessment structures.
**DISCUSSION AND OTHER ELEMENTS**		
**36. INTERPRETATION:** Interpretation anchored to	• Ensure that the discussion interprets the findings in a manner consistent with the evidence generated, considering the CBHPE core components, theoretical	CBHPE is often studied or evaluated without clear evidence of which core components were implemented. Interpreting results

CBHPE core components and principles [1, 2, 19]	frameworks, and existing CBHPE literature.<br>• Interpret findings by Van Melle core component(s) addressed (Primary/Secondary) and clarify which CBHPE principles were actually enacted (e.g., competency-based advancement and promotion vs time-based promotion; tailored learning; programmatic assessment aggregation).<br>• Explicitly link interpretations to the proximal vs intermediate vs distal outcomes reported and the time horizon (i.e., what claims are supported now vs what remains unknown).	using an explicit core-components framework improves conceptual fidelity, prevents overstatements of findings or interpretations, and supports cross-study comparisons of which aspect of CBHPE was actually tested.
**37. MECHANISMS:** Mechanisms of CBHPE impact and explanation of variation [4, 14, 15, 19, 46]	• Explain why outcomes occurred (or did not) by integrating: (1) implementation outcomes (acceptability/feasibility/adoption/reach), (2) fidelity findings, (3) major adaptations, and (4) contextual drivers (e.g., assessor availability, clinical opportunity structure, platform constraints).<br>• Identify the most plausible mechanism(s) and where evidence is strong vs speculative.	For complex interventions, outcome patterns are often only interpretable when paired with information about implementation, mechanisms, and context. This explanatory layer helps distinguish intervention failure from implementation failure and supports transferable learning beyond a single site.
**38. BARRIERS AND FACILITATORS:** Implementation determinants [19, 22, 46, 82-84]	• Report key barriers/facilitators.<br>• If possible, organize them using a determinant framework (e.g., CFIR domains/constructs, Active Implementation Framework) [22, 46, 82].<br>• Connect determinants to the observed implementation outcomes (e.g., which barriers plausibly drove low reach or fidelity).	Determinant frameworks provide a consistent structure for describing barriers/facilitators and improve the usefulness of lessons learned across settings. This also strengthens causal interpretation by linking context/determinants to implementation outcomes.
**39. TRANSFERABILITY AND SCALE:** Implications for scaling and transferability [19, 28, 56, 64, 85, 86]	• State what is likely transferable and under what conditions by identifying core (non-negotiable) vs adaptable elements, and linking these to the observed fidelity and adaptations.<br>• Describe resource requirements and what would need to change for broader use (faculty time, assessor workforce, infrastructure). When implementation occurred across varied sites, discuss how results differed by site/resource context (including low-resource/low/middle income countries-relevant constraints when applicable) and what that implies for spread.<br>• As a way to keep this concise and systematic, authors may organize these implications using a dissemination/implementation lens that prompts attention to who was reached, where adoption occurred, how implementation varied, and whether maintenance is plausible.<br>• Any lessons learned would also be valuable to share.	Decisions about dissemination and scale depend on external validity and feasibility, not effectiveness alone. Readers need to know who the innovation reached, whether settings adopted it, how consistently it was implemented (including fidelity and adaptations), and whether it can be maintained over time. Frameworks commonly used in dissemination/implementation research (e.g., RE-AIM and related expansions) were developed to encourage consistent reporting across these domains and to make results more actionable for real-world adoption and sustainability decisions. Explicitly addressing resource requirements and context-driven variation supports responsible transfer across settings, including resource-diverse environments, by clarifying the conditions under which similar reach, adoption, implementation quality, and sustainment are plausible.
**40. LIMITATIONS and 41. REFLEXIVITY STATEMENT** [2, 35, 87-89]	• Discuss limitations across: (a) internal validity (design, bias, missing data, measurement quality/decision-rule defensibility), (b) implementation limitations (fidelity measurement limits, adaptation drift, uneven rater participation), and (c) external validity (single-site context specificity; limits on transferability).<br>• Explicitly note which outcomes were not observable given the time horizon (e.g., intermediate or distal outcomes).<br>• Add a statement identifying how the research team's background, etc., may impact the CBHPE study findings and work.	Complex, context-sensitive interventions require transparent reporting of limitations to avoid misattributing effects and prevent overgeneralization. Explicitly addressing design and implementation limitations improves interpretability and supports responsible synthesis across studies.
**CONCLUSIONS**		
**42. SUMMARY OF INSIGHTS: 42A. INTERVENTION / IMPLEMENTATION INSIGHTS and 42B. QUALITATIVE INSIGHTS:** Summarize alignment with CBHPE principles and what was	**Intervention/Implementation:**<br>• Summarize (a) which CBHPE components were implemented (ideally using the same Van Melle Primary/Secondary mapping reported earlier(2)), (b) the most important outcomes observed, explicitly distinguishing proximal vs intermediate vs distal and the time horizon, and (c) the headline implementation signal (e.g., feasibility/reach and whether fidelity was sufficient to support conclusions).<br>**Qualitative:**<br>• Summarize the CBHPE-related central themes/constructs/relationships identified.	CBHPE innovations are complex and frequently labeled competency-based, without clear evidence of what was implemented. A structured summary that aligns claims with implemented components and observed outcomes improves interpretability and cross-study comparability and reduces over-claiming when distal outcomes are not yet observable.

actually implemented [1, 2, 19]	If theory-generating, then state the proposed conceptual model/mechanisms (or what is newly clarified) and what remains uncertain.	
**43. PRACTICE IMPLICATIONS:** Actionable implications for practice and implementation [11, 19, 28, 56, 64, 85, 86]	• State 1–3 concrete implications for programs considering adoption: core (non-negotiable) elements vs adaptable elements, minimum infrastructure/workforce needs, and conditions under which similar outcomes are plausible (e.g., supervision model, assessor capacity, platform requirements).<br>• If multi-site/resource-diverse, note whether implementation performance differed by context and what that implies for spread.<br>• (Optional: briefly organize these implications using a dissemination/ implementation lens that prompts attention to who was reached, where adoption occurred, how implementation varied, and whether maintenance is plausible.)	Decisions about spreading educational innovations depend on more than effectiveness; they require information about feasibility, adoption, implementation quality, and sustainability under real-world conditions. Dissemination/implementation frameworks were developed to support systematic reporting and more actionable guidance for adoption and sustainment decisions across settings.
**44. FUTURE RESEARCH:** Recommend next steps for research and evaluation [11, 19, 28, 56, 64, 85, 86]	Specify the next evaluation step(s) needed to strengthen evidence: longer follow-up for distal outcomes, comparative or theory-informed evaluation where feasible, improved measurement/decision-rule evidence (if applicable), and targeted study of implementation questions raised (e.g., drivers of variable reach/fidelity, effects of major adaptations, resource requirements).	For complex interventions, cumulative learning requires clarity about what remains unknown (especially distal outcomes) and what evaluation is needed next. Explicit next-step recommendations accelerate knowledge building across settings and support more rigorous, interpretable evidence over time.

APPENDIX II. CHANGES IN REPORTING GUIDELINES

Phase II: Initial Items from Discussion	Phase III: Revised Items Steering Committee	Phase IV: Revisions from Writing Group, Town Halls, Pilot-Testing, and External Individuals[a]	Final Checklist Items	
			Item	Descriptor
**Title and Abstract**				
Identify the paper as reporting on a CBE initiative or evaluation.	**REVISED:** Identification: Identify the work as a competency-based health professions education (CBHPE) initiative in the title or first line of the abstract, including learner level and setting.	**REVISED:** Identification: Identify the work as a competency-based health professions education (CBHPE **or the discipline-appropriate acronym, such as CBME, CBPE, CBVE, etc.**) initiative in the title **AND abstract**, including learner level and setting.	1. Identification	Identify the work as a competency-based health professions education (CBHPE or the discipline-appropriate acronym, such as CBME, CBPE, CBVE, etc.) or initiative in the title AND abstract, including learner level and setting.
	**NEW:** Study Type: Specify whether the work is an evaluation study and report the study design (e.g., mixed methods, observational, quasi-experimental).	**REVISED:** Study Type: Specify whether the work is an evaluation of a **CBHPE** study and report the study design (e.g., mixed methods, observational, quasi-experimental, **qualitative**).	2. Study Type	Specify whether the work is an evaluation of a CBHPE study and report the study design (e.g., mixed methods, observational, quasi-experimental, qualitative).
Include core CBE components addressed.	**REVISED:** CBHPE Core Components: Identify which Van Melle CBHPE core components were addressed and indicate the degree of implementation (planned/partial/full).	**NO CHANGE**	3. CBHPE Core Components	Identify which CBHPE core components were addressed and indicate the degree of implementation (planned/partial/full).
	**NEW:** Context and Scope: Describe the setting (single vs multi-site), participant groups, scope (pilot vs program-wide), and duration.	**REVISED:** Context and Scope: Describe the **CBHPE** setting (single vs multi-site), participant groups, scope (pilot vs program-wide), and duration.	4. Context and Scope	Describe the CBHPE setting (single vs multi-site), participant groups, scope (pilot vs program-wide), and duration.
	**NEW:** Outcomes and Time Horizon: State the primary outcome(s) and specify whether outcomes are short-term or long-term, including the follow-up period.	**REVISED:** **Study Purpose:** State the primary outcome(s)**/study purpose related to CBHPE** and, **if applicable,** specify whether outcomes are **proximal (in-training), intermediate (transition to practice), or distal (in practice).**	5. Study Purpose	State the primary outcome(s)/study purpose related to CBHPE and, if applicable, specify whether outcomes are proximal (in-training), intermediate (transition to practice), or distal (in practice).
	**NEW:** Implementation: Report key implementation outcomes (e.g., feasibility, reach, adoption) and provide a brief fidelity assessment.	**REVISED:** Implementation or Other Outcomes: Report key implementation elements (e.g., feasibility, reach, adoption) **and/or other outcomes (e.g., clinical, educational, social/behavioral) associated with CBHPE.** ~~**and provide a brief fidelity assessment.**~~	6. Fidelity and Study Outcomes: 6A. Fidelity Evaluation	Provide a brief fidelity evaluation, i.e., implementation delivered as intended, if a CBHPE implementation study.
			6B. Implementation or Other Intervention Outcomes	Report key implementation elements (e.g., feasibility, reach, adoption) and/or other outcomes (e.g., clinical, educational, social/behavioral) associated with CBHPE.
		**REVISED:** **Fidelity evaluation:** Provide a brief fidelity evaluation **(i.e., implementation delivered as intended), if a CBHPE implementation study.**	6C. Summary of Key Insights	Summarize the CBHPE-related central themes/constructs/relationships identified
		**NEW:** **Summary of key insights: Summarize the CBHPE-related central**	6A. Implementation	Report key implementation elements (e.g., feasibility, reach, adoption) and/or

		themes/constructs/relationships identified	or Other Intervention Outcomes	other outcomes (e.g., clinical, educational, social/behavioral) associated with CBHPE.
	**NEW:** Conclusions: Provide conclusions aligned with the evidence and time horizon, including at least one practical implication.	**REVISED:** Conclusions: Provide **CBHPE-related conclusions aligned with the evidence.** ~~and time horizon, including at least one practical implication.~~	7. Conclusions	Provide CBHPE-related conclusions aligned with the evidence.
		**NEW: Key Words: Utilize "Competency-Based Health Professions Education" (CBHPE) as a key word. Supplement with any discipline-specific iterations of CBE as appropriate.**	8. Key Words	Utilize "Competency-Based Health Professions Education" (CBHPE) as a keyword. Supplement with any discipline-specific iterations of CBE as appropriate.
**Introduction**				
Rationale: Describe the problem or gap the CBE initiative is addressing.	**REVISED:** Problem/Rationale: Describe the educational, organizational, and/or societal problem addressed and explain why a CBHPE approach was selected.	**REVISED:** Problem/Rationale: **Position the study in the context of the current CBHPE literature. Describe the problem, hook, gap addressed,** and explain why a CBHPE approach was selected.	9. Problem / Rationale	Position the study in the context of the current CBHPE literature. Describe the problem, hook, gap addressed, and explain why a CBHPE approach was selected.
Discuss Van Melle Core Components	**REVISED:** Conceptual Framing: Anchor the innovation to Van Melle's CBHPE core components and state which components are emphasized.	**NO CHANGE**	10. Conceptual Framing	Anchor the innovation to CBHPE core components and state which components are emphasized.
Objectives: Clearly articulate what the CBE initiative aimed to achieve.	**REVISED:** Objectives: Clearly state the primary and secondary objectives and indicate whether the focus is effectiveness, implementation, or both.	**REVISED:** Objectives Clearly state the primary and secondary objectives of the study and indicate whether the focus is **CBHPE** effectiveness, implementation, **or outcomes evaluation (e.g. readiness to implement CBHPE, fidelity of implementation, assessment program validity, educational outcomes, clinical outcomes).**	11. Objectives	Clearly state the primary and secondary objectives of the study and indicate whether the focus is CBHPE effectiveness, implementation, or outcomes evaluation (e.g., readiness to implement CBHPE, fidelity of implementation, assessment program validity, educational outcomes, clinical outcomes).
	**NEW:** Definitions: Provide operational definitions for key CBHPE terms used in the manuscript.	**REVISED:** Definitions: Reference operational definitions for key CBHPE terms. **If possible, provide definitions for key and any undefined terms used in the manuscript as a glossary/appendix.**	12. Definitions	Reference operational definitions for key CBHPE terms. If possible, provide definitions for key and any undefined terms used in the manuscript as a glossary/appendix.
**Methods**				
	**NEW:** Program Theory: Describe the program theory or theory of change, including assumptions, mechanisms, and causal pathways.	**REVISED: Theoretical Framework & Role in Study: Describe the theoretical framework and its function in the study. Specify whether it is used to:<br>(a) justify/design the CBHPE intervention (e.g., theory of change, program theory, implementation framework, assumptions/mechanisms), and/or<br>(b) guide the inquiry/analysis/interpretation (e.g., conceptual lens, epistemological stance, analytic framework).**	13. Theoretical Framework & Role in Study	Describe the theoretical framework and its function in the study. Specify whether it is used to:<br>(a) justify/design the CBHPE intervention (e.g., theory of change, program theory, implementation framework, assumptions/mechanisms), and/or (b) guide the inquiry/ analysis /interpretation (e.g., conceptual lens, epistemological stance, analytic framework).
Context and Setting (include rich/detailed descriptions of	**REVISED:** Context and Setting: Describe the institutional, educational, regulatory,	**NO CHANGE**	14. Context and Setting	Describe the institutional, educational, regulatory, and/or clinical context,

context)<br>• Describe the institutional/educational context.	and clinical context, including the pre-CBHPE baseline state.			including the pre-CBHPE baseline state.
• State the learner level and scope of implementation.	**REVISED:** Learner Level and Scope: Specify learner stage, number of learners, duration of exposure, and scope of implementation.	**REVISED:** Learner Level ~~and Scope~~**:** Specify learner stage and number of learners.	15. Learner Level	Specify learner stage and number of learners.
		**REVISED:** Scope **of Implementation: Describe the** scope of **CBHPE** implementation **(pilot vs program-wide; duration/time window).**	16. Scope of Implementation	Describe the scope of CBHPE implementation (pilot vs program-wide; duration/time window).
	**NEW:** Stakeholder Role Changes: Describe intended changes in behaviors and roles of learners, faculty, preceptors, and administrators, and how uptake was assessed.	**REVISED:** Stakeholder Role Changes: **Learners:** Describe intended changes in behaviors **and/or** roles of learners **associated with CBHPE and how these were developed,** and how uptake was assessed.	17A. Learners	Describe intended changes in behaviors and/or roles of learners associated with CBHPE and how these were developed, and how uptake was assessed.
		**REVISED:** Stakeholder Role Changes: Others: Describe intended changes in behaviors **and/or** roles of faculty, preceptors, and administrators **associated with CBHPE, how these were developed,** and how uptake was assessed.	17B. Others	Describe intended changes in behaviors and/or roles of faculty, preceptors, and administrators associated with CBHPE, how these were developed, and how uptake was assessed.
	**NEW:** Resources and Constraints: Report resource context relevant to CBHPE feasibility (e.g., faculty availability, infrastructure, time, cost).	**REVISED:** Resources and Constraints: Report resource context relevant to CBHPE feasibility (e.g., **regulatory/accreditation drivers,** faculty availability, infrastructure, time, cost).	18. Resources and Constraints	Report resource context relevant to CBHPE feasibility (e.g., regulatory/accreditation drivers, faculty availability, infrastructure, time, cost).
CBE Components Addressed (mapped to Van Melle's framework)	**REVISED:** CBHPE Core Components Mapping: Map intervention elements to Van Melle's core components (Primary/Secondary/Not addressed).	**REVISED:** Map intervention elements to **CBHPE** core components. ~~(Primary/Secondary/Not addressed)~~	19. CBHPE Core Components Mapping	Map intervention elements to CBHPE core components.
1) Outcome competencies: Describe how outcomes were selected and aligned with frameworks related to discipline (CanMeds, ACGME, etc)	**REVISED:** Outcome Competencies: Describe how competencies were selected, defined, aligned to frameworks, and made transparent.	**NO CHANGE**	19.1. Outcome Competencies	Describe how learner competencies were selected, defined, aligned, and made transparent.
2) Sequenced progression: Detail how progression of competencies is structured (e.g., milestones, levels, developmental rubrics).	**REVISED:** Sequenced Progression: Describe the developmental progression model, milestones or levels, decision points, and calibration practices.	**REVISED:** Sequenced Progression: Describe the developmental progression model, **EPAs/**milestones or levels, decision points, calibration practices, **and/or decision-makers (ex: competency committee).**	19.2. Sequenced Progression	Describe the developmental progression model, EPAs/milestones or levels, decision points, calibration practices, and/or decision-makers (ex: competency committee).
3) Tailored learning: Report on individualized learning plans, flexibility in pacing, or learning pathways.	**REVISED:** Tailored Learning: Describe mechanisms for individualized learning, coaching, pacing flexibility, and learner agency.	**NO CHANGE**	19.3. Tailored Learning	Describe mechanisms for individualized learning, coaching, pacing flexibility, and learner agency.
4) Instructional methods: Describe teaching strategies tailored to support competency development.	**REVISED:** Instructional Methods: Describe teaching and coaching strategies intentionally aligned with competency development.	**NO CHANGE**	19.4. Instructional Methods	Describe teaching and coaching strategies intentionally aligned with competency development.

5) Programmatic assessment: Explain how data were collected longitudinally across tools and settings. Include the use of formative and summative assessments. Describe entrustment, decision-making models, and faculty development.	**REVISED:** Programmatic Assessment: Describe the assessment system, including tools, frequency, data aggregation, entrustment decisions, and assessor development.	**NO CHANGE**	19.5. Programmatic Assessment	Describe the assessment system, including tools, frequency, data aggregation, entrustment decisions, and assessor development.
	**NEW:** Implementation Stages: Describe implementation strategies, governance, training, stakeholder engagement, and rollout process.	**NO CHANGE**	20. Implementation Strategies	Describe implementation strategies, governance, training, stakeholder engagement, and rollout process.
	**NEW:** Fidelity Monitoring: Specify fidelity targets, monitoring methods, and planned vs enacted adaptations.	**REVISED:** Fidelity Monitoring: Specify **how** fidelity **was measured/assessed (implemented as intended) of the CBHPE implementation, such as outputs, uptake/adoption,** monitoring methods, and planned vs enacted adaptations.	21. Fidelity Monitoring	Specify how fidelity was measured/assessed (implemented as intended) of the CBHPE implementation, such as outputs, uptake/adoption, monitoring methods, and planned vs enacted adaptations.
Evaluation Design / Evaluation Plan<br>• For research projects: Study design: observational, mixed methods, intervention, etc.; Data collection tools and validation.; Stakeholder involvement.<br>• For innovations: how might it be evaluated going forward?	**REVISED:** Evaluation Design: Describe the evaluation design, data sources, measures, analytic approach, and stakeholder involvement.	**REVISED:** Evaluation Design: Describe the **CBHPE** evaluation design (**e.g., rapid evaluation, realist evaluation, logic model, quantitative, qualitative, mixed methods**), data sources, measures, analytic approach, and stakeholder involvement.	22. Evaluation Design	Describe the CBHPE evaluation design (e.g., rapid evaluation, realist evaluation, logic model, quantitative, qualitative, mixed methods), data sources, measures, analytic approach, and stakeholder involvement.
	**REVISED:** Evaluation Focus: Specify whether the evaluation focuses on implementation, educational outcomes, or both.	Evaluation Focus: Specify whether the **CBHPE** evaluation focuses on implementation, educational outcomes, or **other elements (e.g., Readiness to Implement CBHPE, Fidelity of Implementation, Validity of the Program of Assessment, Educational Outcomes, Clinical Outcomes).**	23. Evaluation Focus	Specify whether the CBHPE evaluation focuses on implementation, educational outcomes, or other elements (e.g., Readiness to Implement CBHPE, Fidelity of Implementation, Validity of the Program of Assessment, Educational Outcomes, Clinical Outcomes).
	**REVISED:** Future Evaluation: For early-phase innovations, describe the planned evaluation approach and CQI integration.	**REVISED:** Future Evaluation: For early-phase innovations, describe the **CBHPE** planned evaluation approach and CQI integration.	24. Future Evaluation (Recommended not Required)	For early-phase CBHPE innovations, describe the planned evaluation approach and CQI integration.
**Results**				
	**REVISED:** Fidelity Results: Report fidelity across adherence, dose, quality, engagement, and differentiation from baseline.	**REVISED:** Fidelity Results: Report **CBHPE** fidelity **(implemented as intended), such as** adherence**, level of integration,** quality, engagement, and differentiation from baseline.	25. Fidelity Results	Report CBHPE fidelity (implemented as intended), such as adherence, level of integration, quality, engagement, and differentiation from baseline.
Proximal vs distal	**REVISED:** Outcome Timing: Report outcomes by short-term and long-term categories and specify time horizons.	**REVISED:** Outcome Timing: Report **CBHPE-related** outcomes **by time, whether proximal (in-training), intermediate**	26. Outcome Timing	Report CBHPE-related outcomes by time, whether proximal (in-training), intermediate (transition to practice), or

		**(transition to practice), or distal (in practice).**		distal (in practice).
Present implementation outcomes (e.g., acceptability, fidelity, scalability).	**REVISED:** Implementation Outcomes: Report implementation outcomes with denominators and measurement methods.	**REVISED:** ~~**Implementation**~~ Outcomes: Report **CBHPE-related outcomes appropriately based on study design. If quantitative,** denominators and measurement methods **should be included.**	27. Outcomes	Report CBHPE-related outcomes appropriately based on study design. If quantitative, denominators and measurement methods should be included.
	**NEW:** Monitoring Indicators: Report indicators used to monitor outputs and guide continuous quality improvement.	**REVISED:** Monitoring Indicators: Report **CBHPE** indicators used to monitor outputs and guide continuous quality improvement.	28. Monitoring Indicators	Report CBHPE indicators used to monitor outputs and guide continuous quality improvement.
Challenges and adaptations.	**REVISED:** Adaptations: Describe adaptations, timing, rationale, and impact on core versus adaptable elements.	**REVISED:** Adaptations: Describe **CBHPE** adaptations, timing, rationale, and impact on core versus adaptable elements.	29. Adaptations	Describe CBHPE adaptations, timing, rationale, and impact on core versus adaptable elements.
	**NEW:** Contextual Variation: Report implementation performance stratified by site or resource context, where applicable.	**REVISED:** Contextual Variation: Report **CBHPE** implementation **elements** stratified by site or resource context, where applicable.	30. Contextual Variation	Report CBHPE implementation elements stratified by site or resource context, where applicable.
Learner outcomes (progression, entrustment, competence).	**REVISED:** Learner Outcomes: Report learner progression, entrustment decisions, time variability, and remediation/acceleration.	**REVISED:** **Impact on** Learners: Report **CBHPE** impact on learners **and any relevant learner characteristics**. Examples include entrustment decisions **(progression, time variability, and remediation)**, **satisfaction, motivation/engagement.**	31A. Learners	Report CBHPE impact on learners and any relevant learner characteristics. Examples include entrustment decisions (progression, time variability, and remediation), satisfaction, motivation/engagement.
		**NEW:** **Impact on Other Stakeholders: Report CBHPE impact on stakeholders (e.g., faculty, clinician educators, coaches, administrators) and any relevant stakeholder characteristics.**	31B. Other Stakeholders	Report CBHPE impact on other stakeholders (e.g., faculty, clinician educators, coaches, administrators) and any relevant stakeholder characteristics.
Assessment results: triangulation, trends, or meaningful patterns.	**REVISED:** Assessment Synthesis: Describe how assessment data were aggregated and interpreted longitudinally.	**REVISED:** Assessment Synthesis: Describe how **and by whom CBHPE** assessment data were aggregated and interpreted longitudinally.	32. Assessment Synthesis	Describe how and by whom CBHPE assessment data were aggregated and interpreted longitudinally.
Program outcomes	**REVISED:** Program Outcomes: Report program-level outcomes related to progression, assessment burden, and decision processes.	**REVISED:** Program Outcomes: Report **CBHPE** program-level outcomes, **e.g.,** related to progression, changes in assessments, and decision **outcomes/**processes.	33. Program Outcomes	Report CBHPE program-level outcomes, e.g., related to progression, changes in assessments, and decision outcomes/processes.
	**NEW:** Barriers and Unintended Consequences: Report challenges, unintended effects, and mitigation strategies.	**REVISED:** Barriers and Unintended Consequences: Report **CBHPE** challenges, unintended effects, and mitigation strategies.	34. Barriers and Unintended Consequences	Report CBHPE challenges, unintended effects, and mitigation strategies.
	**NEW:** Equity by Design: Describe equity-oriented design features embedded within the CBHPE model.	**REVISED:** Equity by Design: Describe equity-oriented design features embedded within the CBHPE model. **(If not included, use N/A.)**	35. Equity by Design	Describe equity-oriented design features embedded within the CBHPE model. (If not included, use N/A.)
**Discussion and Other**				
Interpret findings in relation to core CBE principles.	**REVISED:** Interpretation: Interpret findings in relation to CBHPE core components, principles enacted, and time horizon.	**REVISED:** Interpretation: **Ensure that the discussion interprets the findings in a manner consistent with the evidence generated, considering the** CBHPE core components, **theoretical frameworks, and existing CBHPE literature.**	36. Interpretation	Ensure that the discussion interprets the findings in a manner consistent with the evidence generated, considering the CBHPE core components, theoretical frameworks, and existing CBHPE literature.
	**NEW:** Mechanisms: Explain plausible	**REVISED:** Mechanisms: Explain plausible	37. Mechanisms	Explain plausible mechanisms of

	mechanisms of impact using implementation, fidelity, adaptation, and context data.	mechanisms of CBHPE impact using **data, such as** implementation, fidelity, adaptation, and context.		CBHPE impact using data, such as implementation, fidelity, adaptation, and context.
Address barriers/facilitators to implementation.	**REVISED:** Barriers and Facilitators: Organize implementation determinants using an established framework (e.g., CFIR).	**REVISED:** Barriers and Facilitators: **Identify CBHPE barriers and facilitators. If possible, use** an established **implementation science** framework (e.g., CFIR) **or theory.**	38. Barriers and Facilitators	Identify CBHPE barriers and facilitators. If possible, use an established implementation science framework (e.g., CFIR) or theory.
Implications for scaling or transferability.	**REVISED:** Transferability and Scale: Discuss core versus adaptable elements, resource requirements, and implications for spread.	**REVISED:** Transferability and Scale: Discuss core versus adaptable elements **of CBHPE**, resource requirements, and implications for **transferability.**	39. Transferability and Scale	Discuss core versus adaptable elements of CBHPE, resource requirements, and implications for transferability.
Discuss limitations (e.g., generalizability, context-specific features).	**REVISED:** Limitations: Discuss design, implementation, and external validity limitations.	**REVISED:** Discuss **CBHPE** design, implementation, and external validity limitations.	40. Limitations	Discuss CBHPE design, implementation, and external validity limitations.
		**NEW: Reflexivity Statement: Add a statement identifying how the research team's background, etc., may impact the CBHPE study findings and work.**	41. Reflexivity Statement [Recommended, not required]	Add a statement identifying how the research team's background, etc., may impact the CBHPE study findings and work.
**Conclusion**				
Summarize alignment with CBE principles.	**REVISED:** Summary of Implementation: Summarize CBHPE components implemented, key outcomes, and implementation signal.	**REVISED:** Summary of **Intervention/**Implementation Insights: Summarize CBHPE components implemented, key outcomes, implementation **adoption, and lessons learned.**	42A. Summary of Intervention / Implementation Insights	Summarize CBHPE components implemented, key outcomes, implementation adoption, and lessons learned.
		**REVISED:** Summary of **Qualitative** Insights: Summarize **the CBHPE-related central themes/constructs/relationships identified. If theory generating, then state the proposed conceptual model/mechanisms (or what is newly clarified), and what remains uncertain.**	42B. Summary of Qualitative Insights	Summarize the CBHPE-related central themes/constructs/relationships identified. If theory generating, then state the proposed conceptual model/mechanisms (or what is newly clarified), and what remains uncertain.
	**NEW:** Practice Implications: State actionable implications for adoption, including minimum requirements and conditions.	**REVISED:** Practice Implications: Describe implications for **CBHPE and potential actionable elements** for adoption, including minimum requirements and conditions.	43. Practice Implications	Describe implications for CBHPE and potential actionable elements for adoption, including minimum requirements and conditions.
Recommend next steps for practice or research.	**REVISED:** Future Research: Recommend next steps for evaluation and evidence building.	**REVISED:** Future Research: Recommend next steps **for CBHPE**, i.e., evaluation and evidence building. **Describe how this study advances CBHPE knowledge.**	44. Future Research	Recommend next steps for CBHPE, i.e., evaluation and evidence building. Describe how this study advances CBHPE knowledge.

[a]Orange color indicates revisions made through iterations with the Writing Group; Green indicates changes made from the Town Hall meetings; Blue indicates changes made from the pilot testing; Purple indicates changes made from the Non-Expert Review; Red indicates changes made from the Peer Review process.

APPENDIX III. CBHPE OUTCOMES REPORTING GUIDELINES: CROSSWALK TO THE CBHPE OUTCOMES THEORY OF CHANGE[19]

	CBHPE Outcomes Theory of Change Element										
**Reporting Guidelines Element**	**CBHPE Core Component**	**Implementation Strategies**	**Drivers of Change**	**Theory of Change**	**Theoretical & Conceptual Foundations**	**Key Stakeholder Actions/ Behavior Changes**	**Implementation Outputs (Early Signals)**	**Outcomes Over Time (Proximal, Intermediate, Distal)**	**Barriers / Enablers**	**Equity Considerations**	**Indicators / Metrics**
**TITLE AND ABSTRACT**											
**1 and 2. IDENTIFICATION and STUDY TYPE**	✓										
**3. CBHPE CORE COMPONENTS**	✓										
**4. CONTEXT AND SCOPE**						✓			✓	✓	
**5. STUDY PURPOSE**											
**6A, 6B, and 6C. IMPLEMENTATION/OTHER OUTCOMES, FIDELITY EVALUATION, AND SUMMARY OF KEY INSIGHTS**								✓			✓
**7. CONCLUSIONS**		✓					✓	✓			✓
**8. KEY WORDS**											
**INTRODUCTION**											
**9. PROBLEM/RATIONALE**			✓	✓	✓						
**10. CONCEPTUAL FRAMING**	✓				✓						
**11. OBJECTIVES**		✓					✓	✓			✓
**12. DEFINITIONS**	✓			✓	✓		✓	✓			
**METHODS**											
**13. THEORETICAL FRAMEWORK & ROLE IN STUDY**	✓	✓	✓	✓	✓	✓	✓	✓	✓	✓	✓
**14. CONTEXT AND SETTING**									✓	✓	✓
**15. LEARNER LEVEL and 16. SCOPE OF IMPLEMENTATION**		✓				✓					
**17. STAKEHOLDER ROLE CHANGES**				✓		✓	✓				✓
**18. RESOURCES AND CONSTRAINTS**		✓							✓		✓
**19. CBHPE CORE COMPONENTS MAPPING**	✓										
**19.1. OUTCOME COMPETENCIES**	✓				✓						✓
**19.2. SEQUENCED PROGRESSION**	✓	✓		✓							✓
**19.3. TAILORED LEARNING**	✓	✓		✓		✓					✓
**19.4. INSTRUCTIONAL**	✓	✓			✓	✓					✓

**METHODS**											
**19.5. PROGRAMMATIC ASSESSMENT**	✓	✓				✓	✓	✓			✓
**20. IMPLEMENTATION STRATEGIES**	✓	✓				✓			✓		✓
**21. FIDELITY MONITORING**	✓	✓					✓				✓
**22. EVALUATION DESIGN, 23. EVALUATION FOCUS, and 24. FUTURE EVALUATION**				✓	✓	✓	✓	✓			✓
**RESULTS**											
**25. FIDELITY RESULTS**	✓	✓					✓				✓
**26. OUTCOME TIMING**								✓			✓
**27. OUTCOMES**		✓					✓				✓
**28. MONITORING INDICATORS**		✓					✓		✓		
**29. ADAPTATIONS**		✓				✓			✓		✓
**30. CONTEXTUAL VARIATION**							✓		✓		✓
**31. IMPACT ON STAKEHOLDERS: 31A. LEARNERS and 31B. OTHER STAKEHOLDERS**	✓							✓			✓
**32. ASSESSMENT SYNTHESIS**	✓						✓	✓			✓
**33. PROGRAM OUTCOMES**											
**34. BARRIERS AND UNINTENDED CONSEQUENCES**							✓	✓	✓	✓	✓
**35. EQUITY BY DESIGN**	✓	✓					✓			✓	✓
**DISCUSSION**											
**36. INTERPRETATION**	✓		✓	✓				✓			
**37. MECHANISMS**			✓	✓	✓				✓		✓
**38. BARRIERS AND FACILITATORS**		✓							✓		✓
**39. TRANSFERABILITY AND SCALE**		✓						✓	✓	✓	✓
**40. LIMITATIONS and 41. REFLEXIVITY STATEMENT**		✓					✓	✓			✓
**CONCLUSION**											
**42. SUMMARY OF INSIGHTS**	✓	✓	✓	✓	✓	✓	✓				
**43. PRACTICE IMPLICATIONS**		✓				✓		✓			
**44. FUTURE RESEARCH:**				✓				✓			✓

REFERENCES

1. Van Melle, E., et al., *A core components framework for evaluating implementation of competency-based medical education programs.* Acad Med, 2019. **94**(7): p. 1002–1009.
2. Hall, A.K., et al., *Outcomes of competency-based medical education: A taxonomy for shared language.* Med Teach, 2021. **43**(7): p. 788–793.
3. Hasson, H., *Systematic evaluation of implementation fidelity of complex interventions in health and social care.* Implement Sci, 2010. **5**: p. 67.
4. Carroll, C., et al., *A conceptual framework for implementation fidelity.* Implement Sci, 2007. **2**: p. 40.
5. Fernandez-Llimos, F., et al., *Improving the quality of publications in and advancing the paradigms of clinical and social pharmacy practice research: The Granada statements.* J Pharm Policy Pract, 2023. **16**(1): p. 43.
6. Lucey, C.R., *Achieving competency-based, time-variable health professions education. Proceedings of a conference sponsored by Josiah Macy Jr. Foundation in June 2017*. 2018, New York, NY: Josiah Macy Jr Foundation.
7. Rhoney, D.H., et al., *The need for competency-based education.* Am J Pharm Educ, 2024. **88**(6).
8. Frank, J.R., et al., *Competency-based medical education: theory to practice.* Med Teach, 2010. **32**(8): p. 638–45.
9. Lingard, L., *Joining a conversation: the problem/gap/hook heuristic.* Perspect Med Educ, 2015. **4**(5): p. 252–253.
10. Hall, A.K., et al., *Approaches to the evaluation of competency-based health professions education implementation from around the globe.* Perspect Med Educ, under review.
11. Cooper, D. and E.S. Holmboe, *Competency-based medical education at the front lines of patient care.* New Engl J Med, 2025. **393**(4): p. 376–388.
12. Martinez-Lage, M., et al., *Implementing the promotion in place model of competency-based time-variable graduate medical education in pathology.* J Grad Med Educ, 2025. **17**(2 Suppl): p. 64–71.
13. Goldhamer, M.E.J., et al., *Promotion in place: A model for competency-based, time-variable graduate medical education.* Acad Med, 2024. **99**(5): p. 518–523.
14. Moore, G.F., et al., *Process evaluation of complex interventions: Medical Research Council guidance.* Bmj, 2015. **350**: p. h1258.
15. Skivington, K., et al., *A new framework for developing and evaluating complex interventions: update of Medical Research Council guidance.* BMJ, 2021. **374**: p. n2061.
16. Hamza, D.M., S. Ross, and I. Oandasan, *Process and outcome evaluation of a CBME intervention guided by program theory.* J Eval Clin Pract, 2020. **26**(4): p. 1096–1104.
17. Smith, J.D., D.H. Li, and M.R. Rafferty, *The Implementation Research Logic Model: a method for planning, executing, reporting, and synthesizing implementation projects.* Implement Sci, 2020. **15**(1): p. 84.
18. Van Melle, E., *Using a logic model to assist in the planning, implementation, and evaluation of educational programs.* Acad Med, 2016. **91**(10): p. 1464.
19. Chen, A.M.H., et al., *The CBHPE Outcomes Theory of Change: An International Consensus Statement on the Mechanisms and Outcomes of Competency-based Education in the Health Professions.* under review.
20. Frye, A.W. and P.A. Hemmer, *Program evaluation models and related theories: AMEE guide no. 67.* Med Teach, 2012. **34**(5): p. e288–99.
21. Cook, D.A., T.J. Beckman, and G. Bordage, *Quality of reporting of experimental studies in medical education: a systematic review.* Med Educ, 2007. **41**(8): p. 737–45.
22. Damschroder, L.J., et al., *Fostering implementation of health services research findings into practice: a consolidated framework for advancing implementation science.* Implement Sci, 2009. **4**: p. 50.
23. Rhoney, D.H., et al., *Behavioral foundations for CBPE: Using the BCW to advance individual readiness to change.* Am J Pharm Educ, 2025. **89**(9): p. 101470.
24. Michie, S., L. Atkins, and R. West, *The behaviour change wheel: A guide to designing interventions*. 2014: Silverback Publishing.
25. Michie, S., M.M. van Stralen, and R. West, *The behaviour change wheel: A new method for characterising and designing behaviour change interventions.* Implement Sci, 2011. **6**(1): p. 42.
26. Simpson, D., et al., *Blueprinting program evaluation evidence through the lens of key stakeholders.* J Grad Med Educ, 2020. **12**(5): p. 629–630.
27. Caverzagie, K.J., et al., *Overarching challenges to the implementation of competency-based medical education.* Med Teach, 2017. **39**(6): p. 588–593.
28. World Health Organization. *Transforming and scaling up health professionals' education and training: World Health Organization Guidelines 2013*. 2013 January 16, 2026]; Available from: https://iris.who.int/server/api/core/bitstreams/64d1ae96-1780-4cf8-a4cb-67fcbc2dbcfd/content.
29. Szulewski, A., et al., *The assessment burden in competency-based medical education: How programs are adapting.* Acad Med, 2023. **98**(11): p. 1261–1267.
30. Thoma, B., et al., *Emerging concepts in the CanMEDS physician competency framework.* Can Med Educ J, 2023. **14**(1): p. 4–12.
31. Holmboe, E.S., et al., *Reflections on the First 2 Years of Milestone Implementation.* J Grad Med Educ, 2015. **7**(3): p. 506–11.
32. Batt, A.M., et al., *CONFERD-HP: recommendations for reporting COmpeteNcy FramEwoRk Development in health professions.* Br J Surg, 2023. **110**(2): p. 233–241.

33. Green, M.L., et al., *Charting the road to competence: developmental milestones for internal medicine residency training.* J Grad Med Educ, 2009. **1**(1): p. 5–20.
34. Andolsek, K.M., et al., *Introduction to the Milestones 2.0: Assessment, Implementation, and Clinical Competency Committees Supplement.* J Grad Med Educ, 2021. **13**(2 Suppl): p. 1–4.
35. Misra, S., et al., *The importance of competency-based programmatic assessment in graduate medical education.* J Grad Med Educ, 2021. **13**(2 Suppl): p. 113–119.
36. Frank, J.R., et al., *Competence By Design: a transformational national model of time-variable competency-based postgraduate medical education.* Perspect Med Educ, 2024. **13**(1): p. 201–223.
37. Wheat, S. and S. Cole, *Individualized learning plans: Who, what, when, where, why, and how?* Ann Fam Med, 2023. **21**(6): p. 560–562.
38. Carraccio, C., A. Lentz, and D.J. Schumacher, *"Dismantling fixed time, variable outcome education: Abandoning 'ready or not, here they come' is overdue".* Perspect Med Educ, 2023. **12**(1): p. 68–75.
39. Hoffman, M., L. Dedow, and J. Boscamp, *Competency-based and less time-bound: a new approach to the macro-structure of a medical school curriculum.* Med Educ Online, 2024. **29**(1): p. 2343205.
40. Romanova, A., et al., *Learning plan use in undergraduate medical education: A scoping review.* Acad Med, 2024. **99**(9): p. 1038–1045.
41. Ericsson, K.A., *Deliberate practice and acquisition of expert performance: a general overview.* Acad Emerg Med, 2008. **15**(11): p. 988–94.
42. Schuwirth, L.W.T. and C.P.M. Van der Vleuten, *Programmatic assessment: From assessment of learning to assessment for learning.* Medical Teacher, 2011. **33**(6): p. 478–485.
43. van der Vleuten, C.P., et al., *A model for programmatic assessment fit for purpose.* Med Teach, 2012. **34**(3): p. 205–14.
44. ten Cate, O., *Entrustability of professional activities and competency-based training.* Med Educ, 2005. **39**(12): p. 1176–7.
45. Ekpenyong, A., et al., *Investigating the roles and impact of clinical competency committees in graduate medical education: A narrative review.* J Grad Med Educ, 2024. **16**(6): p. 662–683.
46. Proctor, E., et al., *Outcomes for implementation research: conceptual distinctions, measurement challenges, and research agenda.* Adm Policy Ment Health, 2011. **38**(2): p. 65–76.
47. Hoffmann, T.C., et al., *Better reporting of interventions: template for intervention description and replication (TIDieR) checklist and guide.* Bmj, 2014. **348**: p. g1687.
48. Hall, A.K., et al., *Evaluating competence by design as a large system change initiative: Readiness, fidelity, and outcomes.* Perspect Med Educ, 2024. **13**(1): p. 95–107.
49. Van Melle, E., et al., *Capturing outcomes of competency-based medical education: The call and the challenge.* Med Teach, 2021. **43**(7): p. 794–800.
50. Dusenbury, L., et al., *A review of research on fidelity of implementation: implications for drug abuse prevention in school settings.* Health Educ Res, 2003. **18**(2): p. 237–56.
51. Dane, A.V. and B.H. Schneider, *Program integrity in primary and early secondary prevention: are implementation effects out of control?* Clin Psychol Rev, 1998. **18**(1): p. 23–45.
52. Ogrinc, G., et al., *SQUIRE-EDU (Standards for QUality Improvement Reporting Excellence in Education): Publication Guidelines for Educational Improvement.* Acad Med, 2019. **94**(10): p. 1461–1470.
53. Craig, P., et al., *Developing and evaluating complex interventions: the new Medical Research Council guidance.* BMJ, 2008. **337**: p. a1655.
54. Bowen, D.J., et al., *How we design feasibility studies.* Am J Prev Med, 2009. **36**(5): p. 452–7.
55. O'Malley, G., T. Perdue, and F. Petracca, *A framework for outcome-level evaluation of in-service training of health care workers.* Hum Resour Health, 2013. **11**: p. 50.
56. Glasgow, R.E., T.M. Vogt, and S.M. Boles, *Evaluating the public health impact of health promotion interventions: the RE-AIM framework.* Am J Public Health, 1999. **89**(9): p. 1322–7.
57. Weiner, B.J., et al., *Psychometric assessment of three newly developed implementation outcome measures.* Implement Sci, 2017. **12**(1): p. 108.
58. Wiltsey Stirman, S., A.A. Baumann, and C.J. Miller, *The FRAME: an expanded framework for reporting adaptations and modifications to evidence-based interventions.* Implement Sci, 2019. **14**(1): p. 58.
59. Iobst, W.F. and E.S. Holmboe, *Programmatic assessment: The secret sauce of effective CBME implementation.* J Grad Med Educ, 2020. **12**(4): p. 518–521.
60. Miller, C.J., et al., *The FRAME-IS: a framework for documenting modifications to implementation strategies in healthcare.* Implement Sci, 2021. **16**(1): p. 36.
61. Kirk, M.A., et al., *Towards a comprehensive model for understanding adaptations' impact: the model for adaptation design and impact (MADI).* Implement Sci, 2020. **15**(1): p. 56.
62. Haley, A.D., et al., *Strengthening methods for tracking adaptations and modifications to implementation strategies.* BMC Med Res Methodol, 2021. **21**(1): p. 133.
63. Safaeinili, N., et al., *CFIR simplified: Pragmatic application of and adaptations to the Consolidated Framework for Implementation Research (CFIR) for evaluation of a patient-centered care transformation within a learning health system.* Learn Health Syst, 2020. **4**(1): p. e10201.
64. Holtrop, J.S., et al., *Understanding and applying the RE-AIM framework: Clarifications and resources.* J Clin Transl Sci, 2021. **5**(1): p. e126.

65. Ten Cate, O., *Nuts and bolts of entrustable professional activities.* J Grad Med Educ, 2013. **5**(1): p. 157–8.
66. Bok, H.G., et al., *Programmatic assessment of competency-based workplace learning: when theory meets practice.* BMC Med Educ, 2013. **13**: p. 123.
67. Schut, S., et al., *Where the rubber meets the road - An integrative review of programmatic assessment in health care professions education.* Perspect Med Educ, 2021. **10**(1): p. 6–13.
68. Goldhamer, M.E.J., et al., *Reimagining the Clinical Competency Committee to enhance education and prepare for competency-based time-variable advancement.* J Gen Intern Med, 2022. **37**(9): p. 2280–2290.
69. Torre, D., et al., *Ottawa 2020 consensus statements for programmatic assessment – 2. Implementation and practice.* Medical Teacher, 2021. **43**(10): p. 1149–1160.
70. de Jong, L.H., et al., *Shaping the right conditions in programmatic assessment: how quality of narrative information affects the quality of high-stakes decision-making.* BMC Med Educ, 2022. **22**(1): p. 409.
71. Wilkinson, T.J. and M.J. Tweed, *Deconstructing programmatic assessment.* Adv Med Educ Pract, 2018. **9**: p. 191–197.
72. Uijtdehaage, S. and L.W.T. Schuwirth, *Assuring the quality of programmatic assessment: Moving beyond psychometrics.* Perspect Med Educ, 2018. **7**(6): p. 350–351.
73. Van Der Vleuten, C.P.M., et al., *Twelve Tips for programmatic assessment.* Med Teach, 2015. **37**(7): p. 641–646.
74. Oswald, A., et al., *Implementing competence committees on a national scale: Design and lessons learned.* Perspect Med Educ, 2024. **13**(1): p. 56–67.
75. Jarrett, J.B., et al., *Evaluating the strengths and barriers of competency-based education in the health professions.* Am J Pharm Educ, 2024. **88**(6): p. 100709.
76. Stoffman, J.M., *Overcoming the barriers to implementation of competence-based medical education in post-graduate medical education: a narrative literature review.* Med Educ Online, 2022. **27**(1): p. 2112012.
77. Jippes, E., et al., *Facilitators and barriers to a nationwide implementation of competency-based postgraduate medical curricula: a qualitative study.* Med Teach, 2012. **34**(8): p. e589–602.
78. Lucey, C.R., et al., *Medical education's wicked problem: Achieving equity in assessment for medical learners.* Acad Med, 2020. **95**(12S Addressing Harmful Bias and Eliminating Discrimination in Health Professions Learning Environments): p. S98–s108.
79. Finn, G.M., J. Tai, and V.D. Nadarajah, *Inclusive assessment in health professions education: Balancing global goals and local contexts.* Med Educ, 2025. **59**(1): p. 88–96.
80. Hennus, M.P. and H.C. Chen, *Equity, diversity, and inclusion in entrustable professional activities based assessment.* Medical Education, 2024. **58**(12): p. 1426–1428.
81. Colbert, C.Y., et al., *Fairness: the hidden challenge for competency-based postgraduate medical education programs.* Perspect Med Educ, 2017. **6**(5): p. 347–355.
82. Blanchard, C., et al., *The Active Implementation Frameworks: A roadmap for advancing implementation of xomprehensive medication management in primary care.* Res Social Adm Pharm, 2017. **13**(5): p. 922–929.
83. Sibicky, S.L., et al., *Enabling factors for the implementation of competency-based curricula in colleges and schools of pharmacy.* Am J Pharm Educ, 2024. **88**(4): p. 100681.
84. Chen, A.M.H., et al., *Examining competency-based education through the lens of implementation science: A scoping review.* Am J Pharm Educ, 2024. **88**(2).
85. Shelton, R.C., D.A. Chambers, and R.E. Glasgow, *An extension of RE-AIM to enhance sustainability: Addressing dynamic context and promoting health equity over time.* Front Public Health, 2020. **8**: p. 134.
86. Gaglio, B., J.A. Shoup, and R.E. Glasgow, *The RE-AIM framework: a systematic review of use over time.* Am J Public Health, 2013. **103**(6): p. e38–46.
87. Holmboe, E.S., *Competency-based medical education and the ghost of Kuhn: Reflections on the messy and meaningful work of transformation.* Acad Med, 2018. **93**(3): p. 350–353.
88. Holmboe, E.S. and J.R. Kogan, *Will any road get you there? Examining warranted and unwarranted variation in medical education.* Acad Med, 2022. **97**(8): p. 1128–1136.
89. Johnson, J.L., D. Adkins, and S. Chauvin, *A review of the quality indicators of rigor in qualitative research.* Am J Pharm Educ, 2020. **84**(1): p. 7120.
